# Quantitative Proteomics Analysis of Lettuce (*Lactuca sativa* L.) Reveals Molecular Basis-Associated Auxin and Photosynthesis with Bolting Induced by High Temperature

**DOI:** 10.3390/ijms19102967

**Published:** 2018-09-28

**Authors:** Jing-Hong Hao, Li-Li Zhang, Pan-Pan Li, Yan-Chuan Sun, Jian-Ke Li, Xiao-Xiao Qin, Lu Wang, Zheng-Yang Qi, Shuang Xiao, Ying-Yan Han, Chao-Jie Liu, Shuang-Xi Fan

**Affiliations:** 1 Beijing Key Laboratory of New Technology in Agricultural Application, National Demonstration Center for Experimental Plant Production Education, Plant Science and Technology College, Beijing University of Agriculture, Beijing 102206, China; haojinghong2013@126.com (J.-H.H.); zll0224@126.com (L.-L.Z.); plee0616@163.com (P.-P.L.); syc546421184@163.com (Y.-C.S.); vipqindada@163.com (X.-X.Q.); 18810986422@163.com (L.W.); 15100258302@163.com (Z.-Y.Q.); jiumingxs@sina.com (S.X.); hyybac@126.com (Y.-Y.H.); cliu@bua.edu.cn (C.-J.L.); 2 Institute of Apicultural Research, Chinese Academy of Agricultural Science, No. 1 Beigou Xiangshan, Beijing 100093, China; apislijk@126.com

**Keywords:** lettuce, bolting, proteome, high temperature, iTRAQ

## Abstract

Bolting is a key process in the growth and development of lettuce (*Lactuca sativa* L.). A high temperature can induce early bolting, which decreases both the quality and production of lettuce. However, knowledge of underlying lettuce bolting is still lacking. To better understand the molecular basis of bolting, a comparative proteomics analysis was conducted on lettuce stems, during the bolting period induced by a high temperature (33 °C) and a control temperature (20 °C) using iTRAQ-based proteomics, phenotypic measures, and biological verifications using qRT-PCR and Western blot. The high temperature induced lettuce bolting, while the control temperature did not. Of the 5454 identified proteins, 619 proteins presented differential abundance induced by high-temperature relative to the control group, of which 345 had an increased abundance and 274 had a decreased abundance. Proteins with an abundance level change were mainly enriched in pathways associated with photosynthesis and tryptophan metabolism involved in auxin (IAA) biosynthesis. Moreover, among the proteins with differential abundance, proteins associated with photosynthesis and tryptophan metabolism were increased. These findings indicate that a high temperature enhances the function of photosynthesis and IAA biosynthesis to promote the process of bolting, which is in line with the physiology and transcription level of IAA metabolism. Our data provide a first comprehensive dataset for gaining novel understanding of the molecular basis underlying lettuce bolting induced by high temperature. It is potentially important for further functional analysis and genetic manipulation for molecular breeding to breed new cultivars of lettuce to restrain early bolting, which is vital for improving vegetable quality.

## 1. Introduction

Bolting is a clear characteristic of the transition from vegetative to reproductive growth in blade root vegetable plants. It is defined as the accelerated and sustained rapid elongation of the stem flower bud differentiation, after which flowering begins. Early bolting refers to the phenomenon of bolting before the formation of the plant product, and seriously affects crop yield and quality. When early bolting occurs, the main characteristics of leaf vegetable performance include the following: premature differentiation of flower buds, reduction in the number of leaves, textural decline, development of a bitter flavor, and the inability to form a compact leaf ball. This process usually causes huge economic losses for producers, and thus strategies to prevent early bolting are urgently needed.

Bolting is regulated by diverse environmental and endogenous factors, such as the temperature, light signals, day length, developmental stage and plant hormones [1]. It consists of a series of physiological and biochemical reactions in plant cells. Researchers have found that the soluble protein, soluble sugar, free amino acid, peroxisome (POD), and vitamin C (Vc) contents are likely germane to the initiation of bolting in mustard and cabbage [2]. Gibberellins (GAs) are phytohormones that regulate many aspects of plant growth and development, including bolting [3]. A puzzling and controversial phenomenon is that ethylene delays bolting in wild-type *Arabidopsis*, yet both the constitutive triple response mutant (*ctr1-1*) and ethylene-insensitive mutants (*etr1-1* and *ein2-5*) exhibit a similar delayed bolting phenotype [4].

In recent studies, several genes and proteins have been implicated in a bolting control according to the isolation of loss-of-function mutants or analysis of transgenic plants. It was found that sugar beet contains a large *CONSTANS*-like gene family, independent of the early-bolting (*B*) gene locus [5]. It was also speculated that the *BrVHA-E1*, *BrSAMS*, *BrrbcL*, and *BrTUA6* genes might be involved in regulating the flower differentiation and bolting of *Brassica rapa* according to their significantly different expression levels [6]. S-adenosylmethionine synthetase (SAMS) is a precursor of ethylene [7]. Zhu et al. found that the serine-62 (Ser-62) phosphorylation of Ethylene Response Factor110 (ERF110) is involved regulating the bolting time of Arabidopsis [8]. Meanwhile, liu et al. found that ETH promotes flowering of Pineapple. It can be speculated that there is a connection between SAMS and bolting, but the specific mechanism of action is not clear. Research by Strompen et al. shows that VHA-E1 is involved in regulating early embryogenesis in Arabidopsis [9]. Zhang et al. speculated that BrVHA-E1 is only associated with the differentiation of flower buds [6]. Microtubules exist widely in the cytoskeleton, and are important for cellular morphology, cell division, transportation, energy transfer, and signal transduction, and α-tubulin (*TUA*) is the basic unit of microtubules. The expression of three *TUAs* was determined in *Brassica rapa*, and the results showed that *BrTUAs* were involved in the regulation of bolting in *Brassica rapa*; *TUAs* affect the speed of shaft elongation [10]. Rubisco is the key regulatory enzyme catalyzing CO_2_ fixation and ribulose diphosphate oxygenase reaction dual function, which determines net photosynthesis [11]. The holoenzyme of Rubisco is composed of eight small subunits encoded by a nuclear multigene family (rbcS), and eight large subunits encoded by a single gene (rbcL) in the multicopy chloroplast genome. Sucrose has been found to directly regulate the expression of the flower regulator LFY [12]. The chloroplast *rbcL* gene encoded Ribulose-1,5-bisphosphate carboxylase oxygenase (RuBisCO), which is the key enzyme involved in the calvin cycle of carbon assimilation during photosynthesis [13]. *BrrbcL* was up-regulated after color change. It boosts the synthesis of photosynthetic products, leading to an increased C/N ratio, and the subsequent occurrence of bolting and flowering in the plant [6]. *ZCE1* is a *cis-cinnamic acid* (*Zusammen*-*cinnamic acid*)-*Enhanced* gene, which encodes a member of the major latex protein-like (*MLPL*) gene family. The *zce1* mutant produced by the RNA-interference technique shows an earlier bolting phenotype in *Arabidopsis*, indicating that *ZCE1* plays a role in delaying bolting [14]. From the perspective of previous researches, many physiological metabolisms and some genes or proteins are related to bolting. However, it is possible that other metabolisms may take part in bolting but are still unknown.

Lettuce (*Lactuca sativa* L.) has economic importance because it is a globally consumed, popular leafy vegetable. This vegetable originated along the Mediterranean coast, and has an optimum growth temperature of 15–20 °C; hence, it is sensitive to high temperatures. When temperatures exceed 30 °C, lettuces undergo early bolting, which lead to decreased nutritional quality, reduced commercial value, and significant losses in productivity and economic benefits. Little is known about bolting in lettuce. It was reported that a high temperature induced bolting in lettuce and that GAs played an important role in this process. *LsGA3ox1* is a gene that is possibly responsible for the increased GA1, but the mechanism of GA metabolism and/or action may differ among cultivars with different bolting characteristics [3]. *SUPPRESSOR OF OVEREXPRESSION OF CO 1* (*SOC1*) encodes a MADS-box protein that integrates multiple flowering signals derived from photoperiod, temperature, hormone and age-related pathways [15]. *SOC1* interacts with multiple MADS-box proteins, including *FRUITFULL* (*FUL*), *AP1* and *AGAMOUS LIKE24* (*AGL24*), and regulates several flowering genes, e.g. by directly binding to their regulatory sequences [16]. Han and colleagues from our laboratory [17] reported that, although GA regulates bolting in lettuce, it may be the MADS-box genes instead, which play a major role in differing the bolting resistance between a bolting resistant line and a bolting sensitive line. A total of 12 MADS-box transcription factors were dramatically induced in lettuce during bolting such as putative *LsSOC1*, *LsAP1*, *LsFUL* and *LsAGL24*.

However, it is unknown whether other hormones and metabolism or main genes/proteins functions in bolting, and the molecular mechanism remains elusive. 

Proteomics is becoming an increasingly important tool to reveal molecular mechanisms at the overall level of protein expression because proteins are directly linked to cellular functions. The mRNA only shows changes at the transcriptional level and cannot fully represent the true level of protein expression. Therefore, proteins and their functions must be investigated to study genetic features. Recently, proteomics has been widely used in the exploration of the resistance mechanism or development characters of plants, such as heat resistance [18], cold resistance and salt resistance [19]. Applying the proteomics approach, the bolting pattern in several plant species has been analyzed, such as *Brassica rapa* [5], *Lactuca sativa* [17], and *Arabidopsis thaliana* [8,14]. Furthermore, the proteomics analysis of the molecular basis of bolting in lettuce (a non-vernalization plant) induced by a high temperature was reported by Han and colleagues from our laboratory [17]. Due to the technological limitation of two-dimensional electrophoresis-based proteomics, only 30 proteins with differential abundance were identified in lettuce in the previous work from our laboratory [17]. The technological advances in the resolution and accuracy of mass spectrometry and efficient labeling quantification of protein abundance levels, allow for a greater depth of proteome coverage. The isobaric tagging for relative and absolute quantification (iTRAQ) was performed. This provides an opportunity for gaining new insight into the molecular basis that drives the bolting of lettuce induced by high temperatures. Here, we revealed potential mechanisms involved in the regulation of bolting by proteomics. The results were verified using Western blot, real-time quantitative fluorescence PCR, and physiological analyses. Finally, a possible pathway map of bolting in lettuce induced by high temperatures was proposed. Our data may potentially be important for resolving the challenging problem of early bolting in vegetable cultivation, the genetic manipulation of lettuce and other bolting plants. 

## 2. Results

### 2.1. The Morphological and Physiological Changes of Lettuce Stems During Bolting Induced by High Temperatures

**Figure 1:**
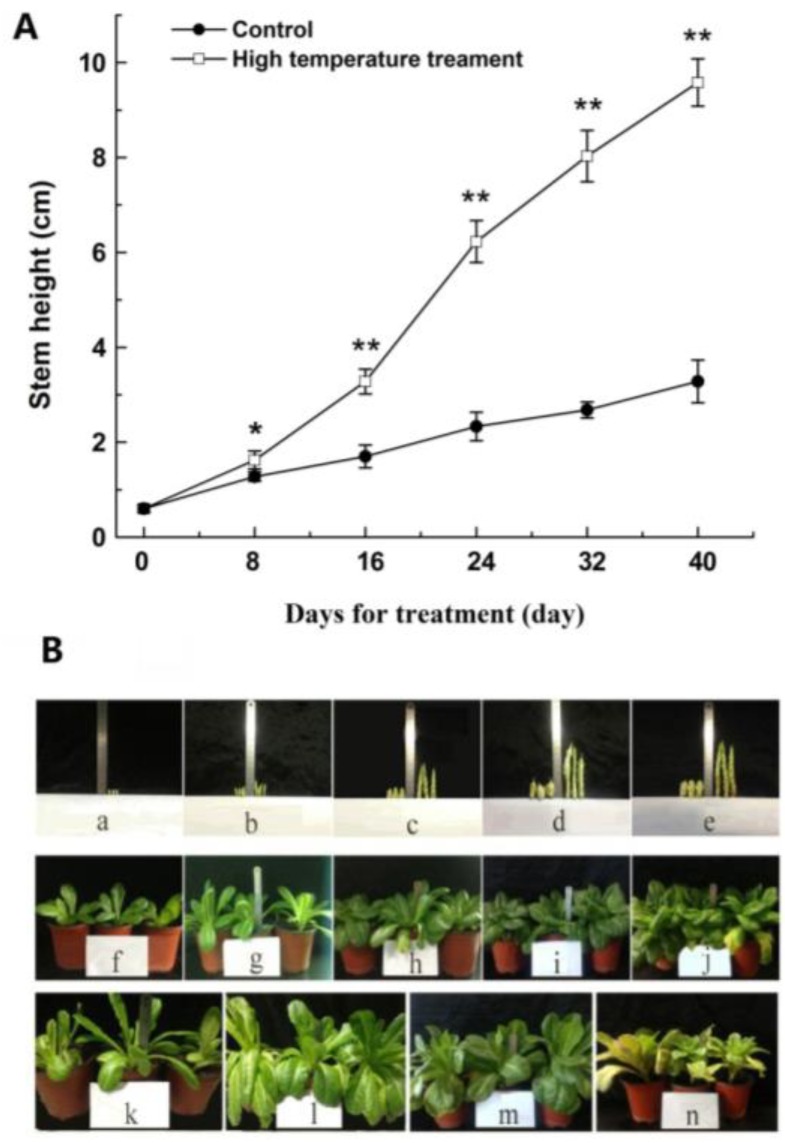
Change of stem height in lettuce after high temperature treatment of 33/25 °C. (**A**) Changes of stem length under 20/13 °C (day/night) (control) and 33/25 °C (day/night) treatment for 40 days. The data (mean ± SD) are the means of three replicates with standard errors shown by vertical bars, *n* = 9. * and ** indicate significant difference at *p* < 0.05 and *p* < 0.01 by Tukey’s test, respectively. (**B**) The phenotypes of lettuce under 20/13 °C (day/night) (control) and 33/25 °C (day/night) treatment for 40 days. (a–e) Stem growth after different temperature treatment for 0, 8, 24, 32 and 40 days (control on left and high temperature treatment on right). Representative images of plants under control (g–j) and high temperature treatment (k–n) for 8, 24, 32 and 40 days, and (f) plant growth at Day 0.

**Figure 2:**
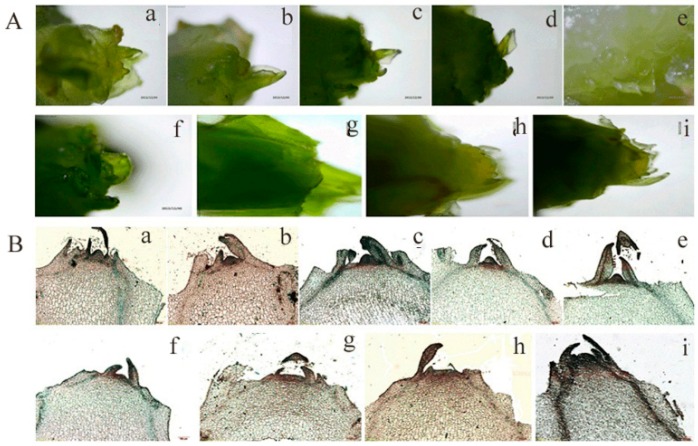
Change of flower bud differentiation of lettuce after high temperature treatment of 33/25 °C. (**A**) Representative images of stem tips under 20/13 °C (day/night) (control) and 33/25 °C (day/night) treatment for 40 days. Representative images of stem tip growth under control (b–e) and high temperature treatment (f–i) for 8, 24, 32 and 40 days, and (a) stem tip growth at Day 0. (**B**) The progress of flower bud differentiation. Representative images of morphology of flower bud under control (b–e) and high temperature treatment (f–i) for 8, 24, 32 and 40 days, and (a) stem tip growth at Day 0.

High temperature treatment promoted stem elongation. On Day 8, the length of the stem had increased, with a significant difference (*p* < 0.05) in the high-temperature group compared to the control group. With a longer treatment time, the stem elongation rate accelerated significantly. On Day 16, a strongly significant increase (*p* < 0.01) of 92.9% in the stem length was observed in the high-temperature group, relative to the control group. Afterwards, the stem increase trend was more significant (Figure 1A). The leaves of the high-temperature group turned yellow and withered on Day 40, whereas the leaves of the control group grew well (Figure 1B). A significant change was observed at the stem tip (Figure 2). As shown in Figure 2, the stem tip in the control group remained conical throughout the entire experimental/observational period. The growing point of the stem tip in the high-temperature group remained conical until Day 8. Subsequently, the growing point became larger and less prominent. On Day 24 of the treatment group, the growing point was completely flattened, and the basal inflorescence was entirely raised. On Day 40, part of the phyllary was differentiated at the base of the inflorescence. Combining the change of stem elongation with the progress of flower bud differentiation under a high temperature, it was concluded that obvious bolting had occurred from Day 8 to Day 40 after treatment with a high temperature. The plants showed obvious bolting on Day 32 under a high temperature, thus Day 32 was selected for sampling for the proteome and physiology analyses.

**Figure 3:**
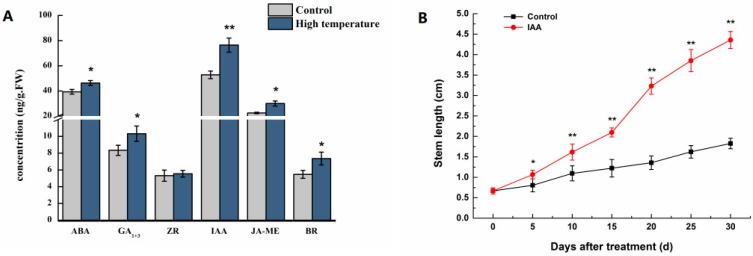
Physiological measurements of lettuce after high temperature treatment of 33/25 °C. (**A**) Content of endogenous hormones (auxin (IAA), gibberellins (GA_1+3_), zeatin (ZR), jasmonic acid methyl ester (JA-ME), abscisic acid (ABA), and brassinosteroid (BR)) in the stems of lettuce in the condition of 20/13 °C (day/night) (control) and 33/25 °C (day/night) treatment for 32 days. (**B**) Stem elongation of lettuce after exogenous 40 mg/L auxin treatment. * and ** indicate significant difference at *p* < 0.05 and *p* < 0.01 by Tukey’s test, respectively.

From the stem tissues of lettuces in control and high-temperature groups, the contents of six endogenous hormones, namely gibberellins (GA_1+3_), zeatin (ZR), brassinosteroid (BR), jasmonic acid methyl ester (JA-ME), auxin (IAA), and abscisic acid (ABA), were examined. As compared to the control group, the contents of ABA, GA_1+3_, ZR, IAA, JA-ME, and BR were significantly higher in the high-temperature group, and there was a stronger significant difference (*p* < 0.01) in IAA than in ABA, GA_1+3_, JA-ME, and BR (*p* < 0.05), with increases of 44.9%, 17.8%, 23.7%, 33.3%, and 34.4%, respectively (Figure 3A).

To determine whether IAA mediates bolting in lettuce, we next explored the effect of exogenous IAA on the bolting of lettuce. As shown in Figure 3B, IAA could promote bolting. In the IAA treated plants, lettuce was obviously extended on Day 5, and the stem length was increased by 32.6% with significant difference (*p* < 0.05). The largest increase ratio was 138.3% on Day 30 compared to mock-treated plants, suggesting that exogenous IAA accelerates bolting.

### 2.2. Identification of Differential Abundance Proteins Using iTRAQ in Lettuce Stems During Bolting Induced by High Temperature

**Figure 4:**
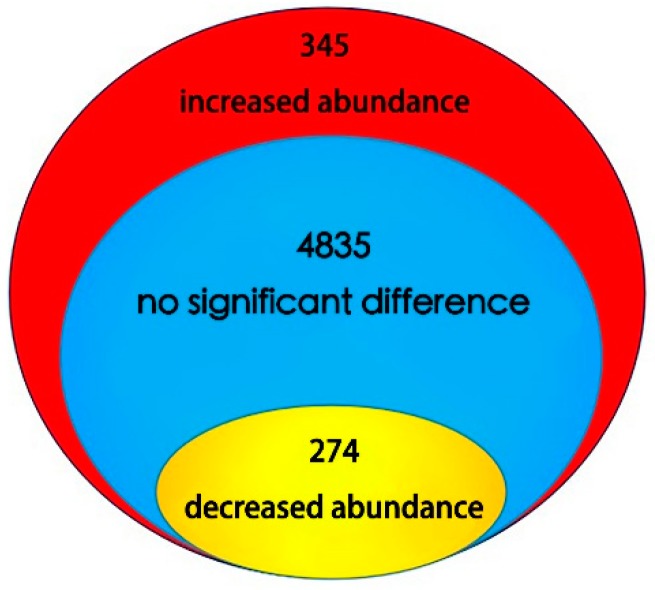
The distribution of proteins with differential abundance.

By means of the iTRAQ-labeled proteomics approach, 5454 proteins were identified in the lettuce stems, as shown in Table S1. The mass spectrometry proteomics data have been deposited in the ProteomeXchange Consortium (http://www.proteomexchange.org) via the PRIDE database, with the dataset identifier, PXD008610. A total of 619 proteins changed significantly in abundance, and 345 of these proteins had increased abundance (red section in Figure 4) while 274 had decreased abundance (yellow section in Figure 4). Detailed information on proteins with differential abundance is shown in Table S2, and the spectra of these proteins, with one unique peptide, are shown in Table S3. Among these, the increased abundance proteins with the highest fold change were hypothetical protein Ccrd_011733 (3.22) and bet v i domain-containing protein (2.51). For the decreased abundance proteins, these were 3-n-debenzoyl-2-deoxytaxol n-benzoyltransferase (0.38), and protein light-dependent short hypocotyls 5-like (0.39). All peptide match information including *m*/*z*, score, delta, PTM. expect value, PSMs, PEP, Charge and RT are shown in Table S4.

### 2.3. Functional Classification and Metabolic Pathways of Differential Abundance Proteins

**Figure 5:**
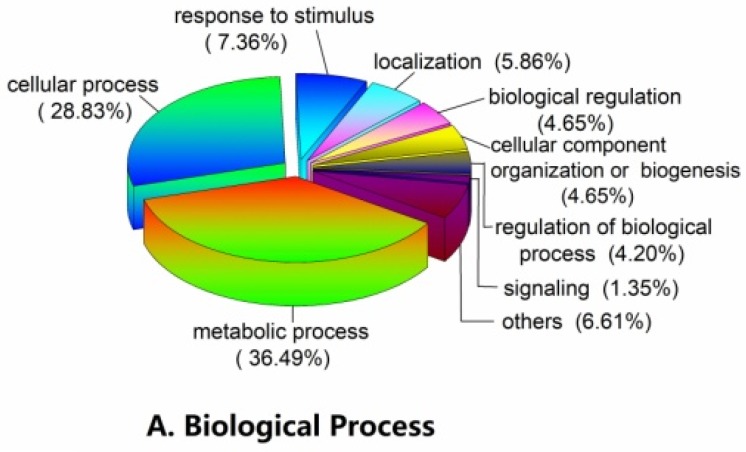

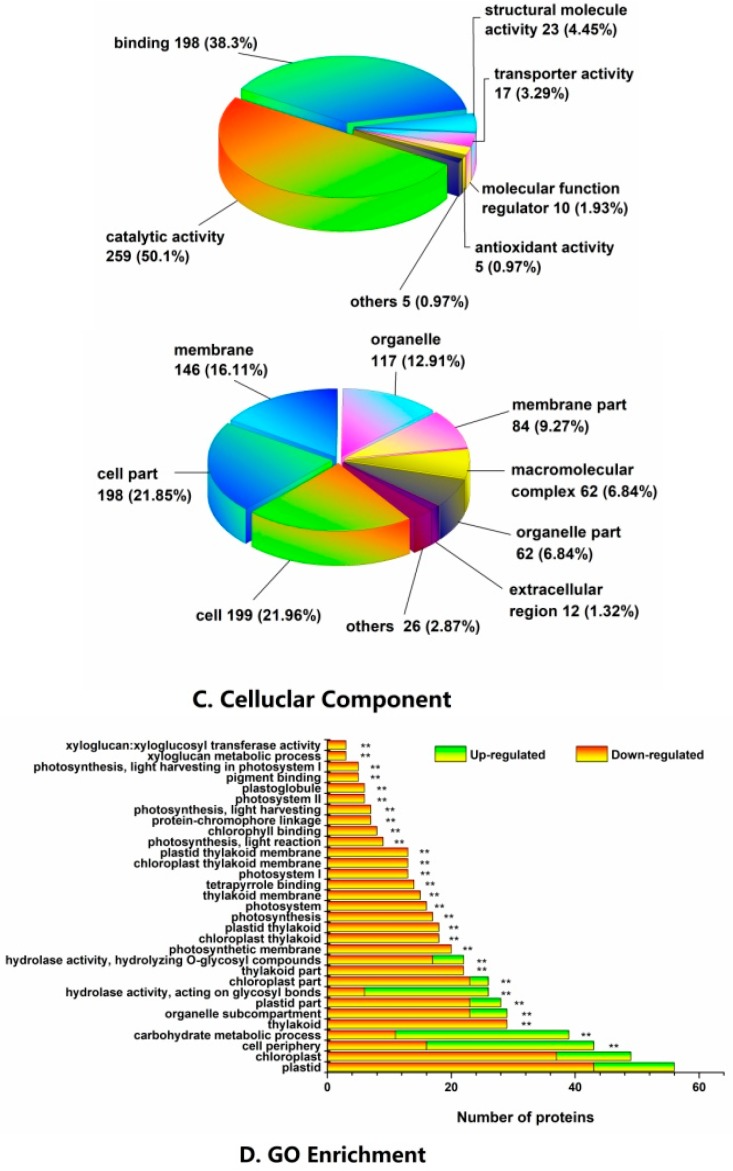
ClueGO and GO enrichment analysis of proteins with differential abundance: (**A**) Biological Process; (**B**) Molecular Function; (**C**) Cellular Component; and (**D**) GO enrichment. ** indicate significant difference at *p* < 0.01 by Tukey’s test, respectively.

We aimed to study the mechanisms by which proteins modulate lettuce bolting induced by high temperatures. Based on the BLAST alignment, Gene Ontology (GO) classification, and literature [20], the identified proteins were classified into 13 functional categories. To identify the significant changes in biological process (BP), molecular function (MF), and cellular component (CC) between the control and high-temperature treatment groups, GO annotation was performed using the Trinotate through BLAST search against the well-annotated protein sequences (SwissProt). Of the total differential proteins, 1796 GO terms were annotated (Figure 5). At level 2, the proteins with differential abundance in bolting under a high temperature in the BP category were annotated with the following terms: Metabolic process (36.49%), cellular process (28.83%), response to stimulus (7.36%), localization (5.86%), biological regulation (4.65%), etc. Similarly, the catalytic activity (50.15%), binding (38.30%), structural molecule activity (4.45%), transporter activity (3.29%), and other (3.87%) terms were annotated in the MF category. In the CC category, cell (21.96%), cell part (21.85%), membrane (16.11%) and organelle (12.91%) terms were annotated. Next, the proteins with increased and decreased abundance were utilized for the GO term enrichment analysis (Figure 5D). Results show that the main GO enrichment functions of increased abundance proteins were: Plastid (43), chloroplast (37), thylakoid (29), cell periphery (26), organelle subcompartment (23), plastid part (23), and chloroplast part (23). The main GO enrichment functions of decreased abundance proteins were carbohydrate metabolic process (28), cell periphery (27), hydrolase activity, acting on glycosyl bonds (20), plastid (13), and chloroplast (12). Overall, most proteins with differential abundance taking part in thylakoid, thylakoid part, photosynthetic membrane, plastid thylakoid, photosynthesis, etc.

**Figure 6:**
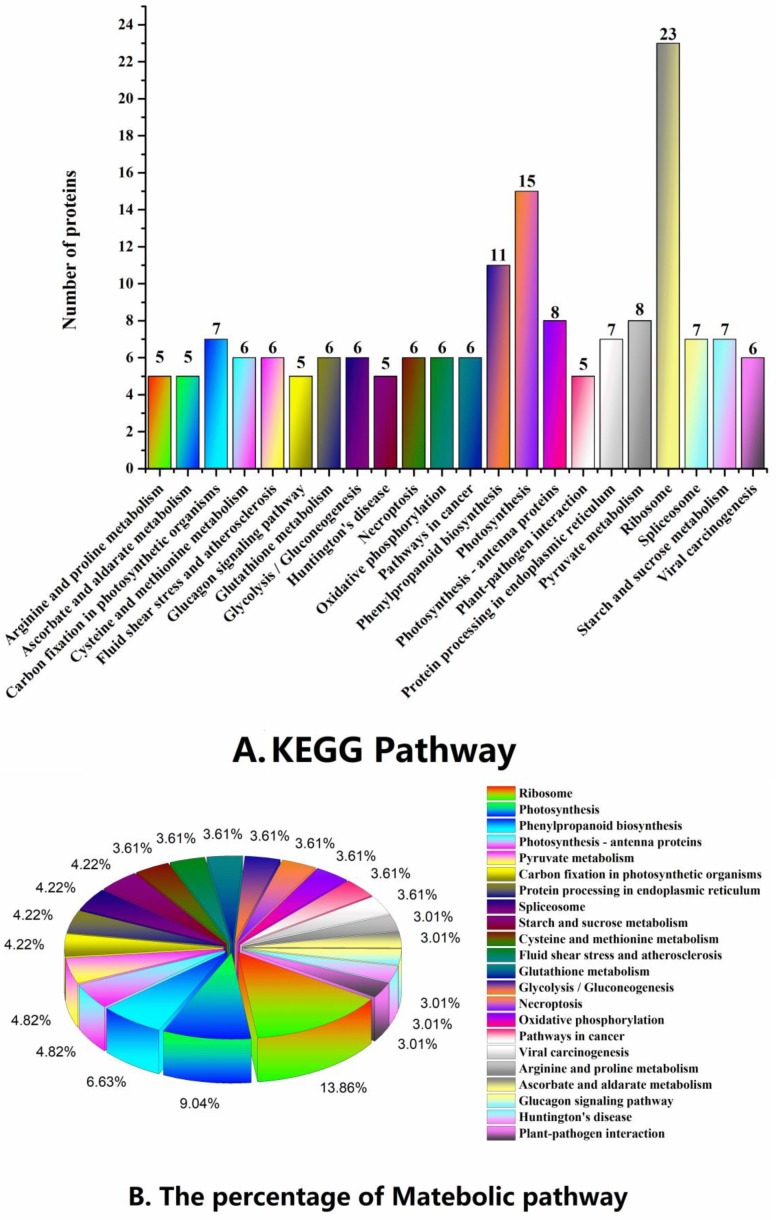

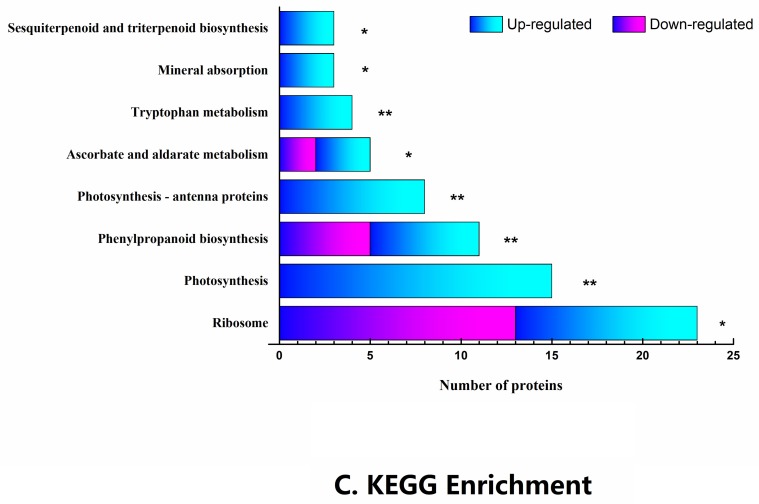
Metabolic analysis of KEGG pathway (matched number more than four) and KEGG Enrichment: (**A**) KEGG Pathway; (**B**) the percentage of Metabolic Pathway; and (**C**) KEGG Enrichment. ^*^ and ^**^ indicate significant difference at *p* < 0.05 and *p* < 0.01 by Tukey’s test, respectively.

To analyze and identify the major metabolic and signal transduction pathways of the proteins with differential abundance, the KEGG database [21] was used. After annotation, the KO number of proteins that were homologous/similar to that of the related KEGG pathway via sequence alignment was determined, and 189 KEGG signaling/metabolic pathways associated with 467 proteins were extracted. Related plants accounted for 76.90% of these proteins. As shown in Figure 6A,B, the metabolic pathways observed in over half of the proteins were: Ribosome (15.03%), photosynthesis (9.80%), phenylpropanoid biosynthesis (7.19%), pyruvate metabolism (5.23%), photosynthesis-antenna proteins (5.23%), starch and sucrose metabolism (4.58%), carbon fixation in photosynthetic organisms (4.58%), protein processing in endoplasmic reticulum (4.58%), spliceosome (4.58%), oxidative phosphorylation (3.92%), glycolysis/gluconeogenesis (3.92%), Cysteine and methionine metabolism (3.92%), Glutathione metabolism (3.92%), and necroptosis (3.92%). Several other significant metabolic pathways were observed as well, such as plant–pathogen interaction (3.27%), peroxisome (2.61%), tryptophan metabolism (2.61%), plant hormone signal transduction (2.61%), and metabolism of xenobiotics by cytochrome P450 (1.96%). To better understand the key metabolic pathways involved in the bolting of lettuce, all differential proteins were successfully enriched and aligned with 14 KEGG pathways (Figure 6C). As shown in Figure 6C, we found that the significantly enriched metabolic pathways of increased abundance proteins were photosynthesis (15), ribosome (10), photosynthesis antenna proteins (8), phenylpropanoid biosynthesis (6), ascorbate and aldarate metabolism (5), tryptophan metabolism (4), mineral absorption (3) and sesquiterpenoid and triterpenoid biosynthesis (3). The significantly enriched metabolic pathways of decreased abundance proteins were ribosome (13), phenylpropanoid biosynthesis (5) and ascorbate and aldarate metabolism (2). Furthermore, all the proteins with differential abundance that take part in photosynthesis, photosynthesis-antenna proteins, tryptophan metabolism, mineral absorption, and sesquiterpenoid and triterpenoid biosynthesis had increased abundance.

**Figure 7:**
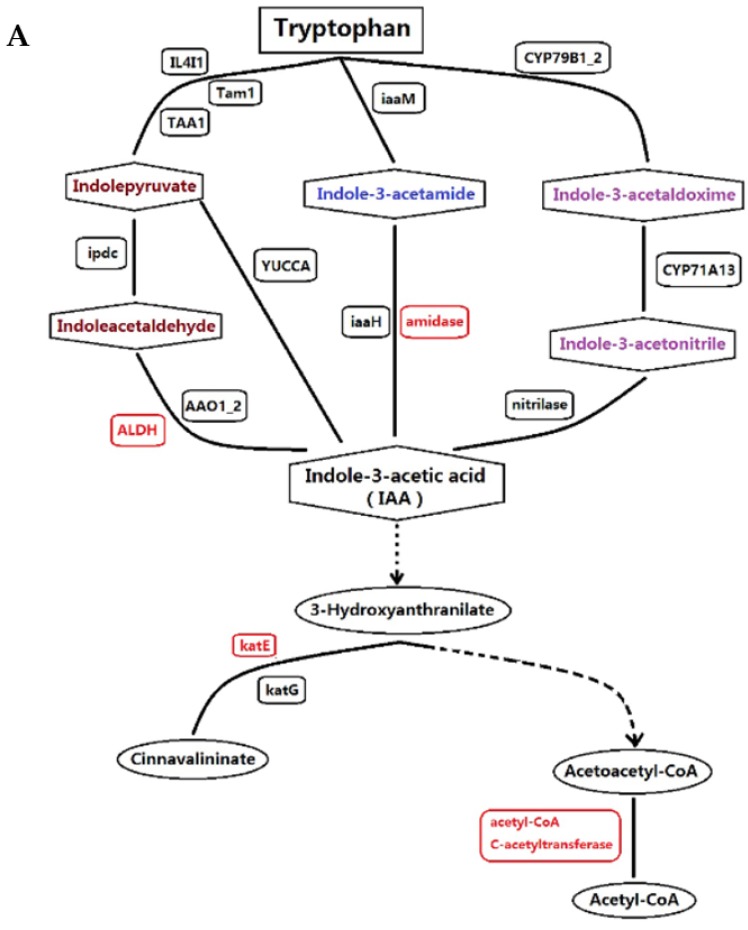

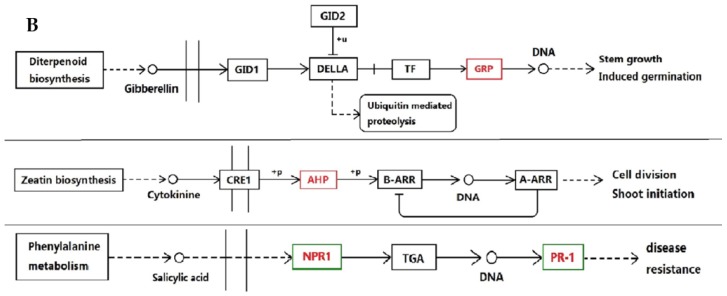
Maps of tryptophan metabolism and plant hormone signal transduction pathways: (**A**) tryptophan metabolism (auxin biosynthesis pathway); and (**B**) plant hormone signal transduction pathway. The red rectangles represent increased abundance proteins, and the green rectangles represent decreased abundance proteins. Definition of proteins indicated in Figure 7A: ALDH, aldehyde dehydrogenase family 2 member mitochondrial; KatE, catalase. Definition of proteins indicated in Figure 7B: GRP, gibberellin regulated protein; AHP, histidine-containing phosphotransfer protein; NPR1, regulatory protein NPR1; PR-1, pathogenesis-related protein 1. The dotted line means many steps of process; The double line means cytomembrane; +u means ubiquitination; +p means Phosphorylation; Vertical lines mean dissociation; The dotted line with a circle means chemical molecule undergoing several steps to combine with next substance.

In the last step of the biosynthesis of indoleacetate in tryptophan metabolism (auxin biosynthesis) (Figure 7A), four proteins with increased abundance were identified: aldehyde dehydrogenase family 2 member mitochondrial (ALDH), amidase family protein, catalase (KatE), and Acetyl-CoA c-acetyltransferase. In the plant hormone signal transduction pathway (Figure 7B), four proteins with differential abundance involved three hormone signal transduction pathways: GRP in gibberellin signal transduction, AHP in cytokinine signal transduction, and NPR1 and PR-1 in salicylic acid signal transduction. Among these pathways, the proteins with increased abundance were GRP and AHP and the proteins with decreased abundance were NPR1 and PR-1.

**Figure 8:**
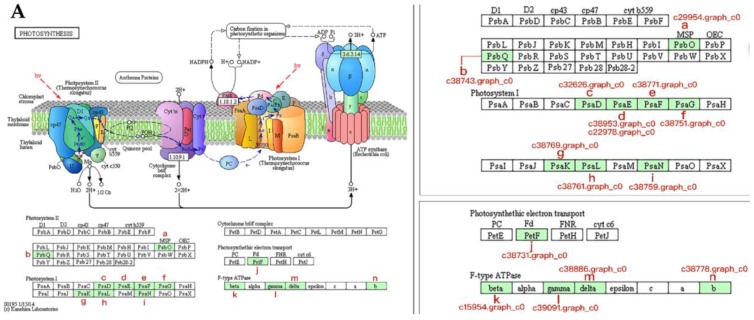

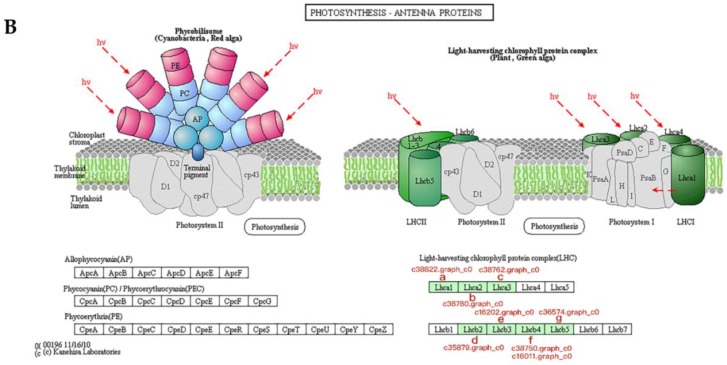
Maps of photosynthesis and photosynthesis-antenna proteins pathways: (**A**) photosynthesis pathway; and (**B**) photosynthesis-antenna proteins pathway. (**A**) The green background rectangles on the left side of the graph represent the identified proteins with differential abundance. The right side of the graph is the enlarged image of the important part, and the proteins with differential abundance are labeled with accession no. (**B**) The green background rectangles represent the identified proteins with differential abundance and labeled with accession no. Definition of proteins indicated in Figure 8A: a: PsbO 1, photosystem II oxygen-evolving enhancer protein 1; b: PsbQ 3, photosystem II oxygen-evolving enhancer protein 3; c: PsaD, photosystem I subunit II; d: PsaE, photosystem I reaction center subunit IV; e: PsaF, photosystem I reaction center subunit III; f: PsaG, photosystem I subunit V; g: PsaK, photosystem I subunit X; h: PsaL, photosystem I subunit XI; i: PsaN, photosystem I subunit PsaN; j: PetF, ferredoxin; k: beta (β), F-type H^+^-transporting ATPase subunit beta; l: gamma (γ), F-type H^+^-transporting ATPase subunit gamma; m: delta (δ), F-type H^+^-transporting ATPase subunit delta; n: b, F-type H^+^-transporting ATPase subunit b. Definition of proteins indicated in Figure 8B: Lhca1–3, light-harvesting complex I chlorophyll a/b binding protein 1–3; Lhcb2–5, light-harvesting complex II chlorophyll a/ binding protein 2–5. alpha (α), F-type H^+^-transporting ATPase subunit alpha; D1: protein subunit D1; D2: protein subunit D2; Arrow: light.

In photosynthesis (Figure 8A) and photosynthesis-antenna proteins (Figure 8B) pathways, there were 14 and 7 proteins with differential abundance, respectively, all of which had increased abundance. In the photosynthesis pathway, the proteins were associated with photosystem I, photosystem II, cytochrome b6/f complex, photosynthetic electron transport, and f-type ATPase (Figure 8A). In the photosynthesis-antenna proteins pathway, the proteins with differential abundance were implicated in allophycocyanin (AP), allophycocyanin (PC)/phycoerythrocyanin (PEC), phycoerythrin (PE), and the light-harvesting chlorophyll protein complex (LHC) (Figure 8B).

### 2.4. Hierarchical Clustering of Protein Profiles

**Figure 9:**
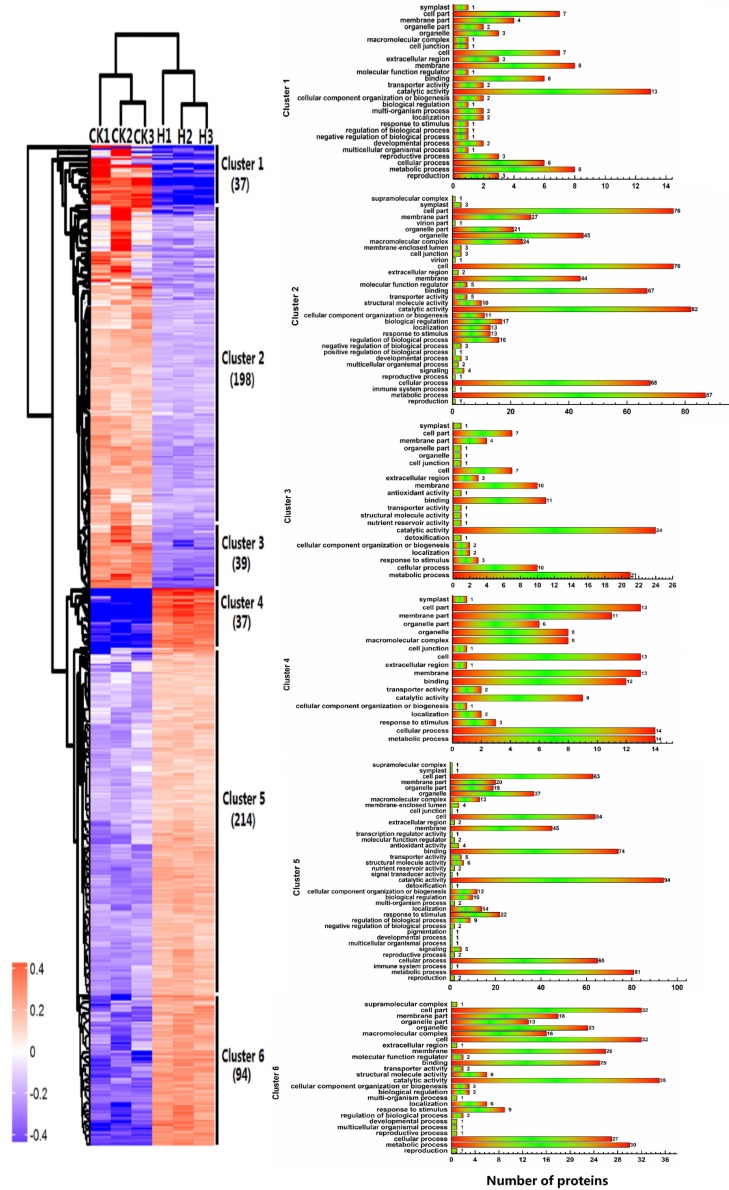
Hierarchical clustering analysis of the differential proteins in lettuce stem under high temperature. The left graph represents hierarchical clustering of proteins with differential abundance. The right graphs represent protein functional classification of the clusters.

To identify the proteins with similar expression patterns, hierarchical clustering was performed. An uncentered correlation was used to define the similarity. The hierarchical clusters were assembled using the average linkage clustering method. The proteins with differential abundance were classified into six main clusters (Figure 9). Compared to the control group, the proteins in Clusters 1–3 of the high-temperature group have increased abundance and the proteins in Clusters 4–6 have decreased abundance. Cluster 1 had the highest increased abundance and included 37 proteins; Cluster 6 had the highest decreased abundance and included 94 proteins. The number of proteins in Clusters 2 and 5 accounted for approximately half of the total number of proteins with differential abundance and consisted of 198 and 214 proteins, respectively, whereas Clusters 3 and 4 consisted of 39 and 37 proteins, respectively. To better visualize the protein clusters during bolting under a high temperature, the significant proteins with differential abundance were also sorted and analyzed by clusters in the form as shown on the right side of Figure 9. After performing a comparison and analysis of the six images, we found that the major biological processes involved in each cluster were as follows: Cluster 1: catalytic activity (13), metabolic process (8), membrane (8), cell (7), cell part (7), cellular process (6) and binding (6); Cluster 2: metabolic process (87), catalytic activity (82), cell (76), cell part (76), cellular process (68), binding (67) and organelle (45); Cluster 3: catalytic activity (24), metabolic process (21), binding (11), cellular process (10), cell (7) and cell part (7); Cluster 4: metabolic process (14), cellular process (14), membrane (13), cell (13), cell part (13) and binding (12); Cluster 5: catalytic activity (94), metabolic process (81), binding (74), cellular process (65), cell (64) and cell part (63); and Cluster 6: catalytic activity (35), cell (32), cell part (32), metabolic process (30), cellular process (27), membrane (26) and binding (25).

### 2.5. Expression Levels of Genes Encoding Some Identified Proteins

**Figure 10:**
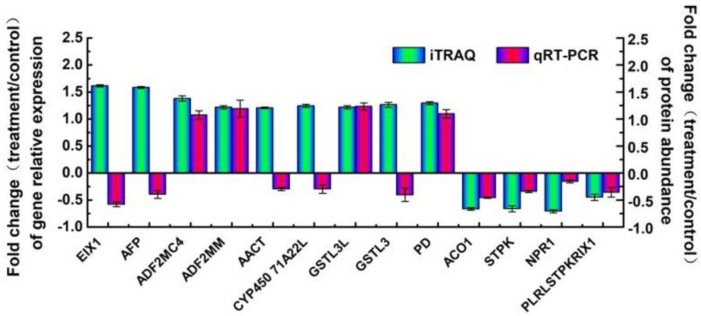
Correlation of mRNA level and protein abundance by iTRAQ. The fold-change of treatment/control in transcript level using the qRT-PCR approach of 19 candidate genes involved in the identified proteins with differential abundance and the protein abundance level by iTRAQ is shown in the figure. The positive number indicates increased abundance, and the negative number indicates decreased abundance. Each histogram represents the mean value of three biological replicates, and the vertical bars indicate the standard error (*n* = 3). Definition of 19 candidate genes involved in the identified proteins with differential abundance: EIX1, esterase isoform x1; AFP, amidase family protein; ADF2MC4, aldehyde dehydrogenase family 2 member c4; ADF2MM, aldehyde dehydrogenase family 2 member mitochondrial; AACT, acetyl-CoA c-acetyltransferase 3; CYP 71A22L, cytochrome p450 71a22-like; GSTL3L, glutathione s-transferase l3-like; GSTL3, glutathione s-transferase l3; PD, phytanoyl-dioxygenase; ACO1,1-aminocyclopropane-1-carboxylate oxidase 1; STPK, serine threonine-protein kinase; NPR1, regulatory protein NPR1; PLRLSTPKRIX1, probable lrr receptor-like serine threonine-protein kinase rfk1 isoform x1.

To understand the relationship between the abundance of a protein and the level of its gene transcripts, we measured the expression profiles of genes encoding 13 selected key node proteins. These proteins are primarily observed in five major functional groups: Phytohormone metabolism (GA, IAA, and ETH), signal transduction, oxido-reduction, ubiquitin degradation, and protein kinase. The mRNA expression trend showed that eight proteins (ADF2MC4, ADF2MM, GSTL3L, PD, ACO1, STPK, NPR1 and PLRLSTPKRIX1) were consistent with the protein abundance (Figure 10). However, the expression levels of five proteins (EIX1, AFP, AACT, CYP71A22 and GSTL3) did not conform to at the mRNA and protein level, which might have been caused by the presence of post-translational modifications.

## 3. Discussion

### 3.1. Proteins Implicated in Hormone Metabolism During Bolting in Lettuce Under a High Temperature

Flower bud differentiation, bolting, and flowering in plants are complex processes. When plants enter the reproductive stage from vegetative growth, nutrients are gradually redistributed to the reproductive organs. This process is the result of the combined effects of various factors. During the whole plant growth and development, including bolting, phytohormones play a critical regulatory role in physiological metabolism and morphogenesis [22]. IAA was the first plant hormone to be discovered. The famous “acid growth theory” shows that, after growing cells are treated with IAA, the pH of the cell wall is reduced, and cell wall relaxation is increased, leading to increased cell elongation. The researchers of this theory found that the primary transcript effect, associated with elevated steady-state auxin concentrations, on elongating root cells, is the up-regulation of cell wall remodeling factors, notably expansins, whereas plant hormone signaling pathways maintain remarkable homeostasis [23]. The *SAUR19* subfamily of *SMALL AUXIN UP RNA* genes promotes cell expansion [24], and the NPH4/auxin response factors ARF 7 and ARF19 promote leaf expansion and auxin-induced lateral root formation [25]. IAA promotes the elongation of stems, whereas auxin deficiencies lead to the inhibition of stem elongation [26,27]. In this study, we found that the following four proteins in the tryptophan metabolism pathway had increased abundance in the last step of the synthesis of indole acetic acid: The aldehyde dehydrogenase family 2 member mitochondrial (ALDH), amidase family protein, catalase (KatE), and Acetyl-CoA c-acetyltransferase. These results suggested that the regulation of IAA for bolting in lettuce may be related to the increased abundance of the above four enzymes, and the specific performance of these proteins affect the biosynthesis of IAA. The induced gene expression was consistent with the increased IAA content in stems subjected to a high temperature (Figure 3). In combination with the finding that exogenous IAA-accelerated bolting (Figure 3), this further suggests that IAA was closely related to bolting. However, applications of IAA and IAA inhibitors in spinach plants had no clear effect on flower bud development and bolting in either treatment. The possible reason for this is that the principle of IAA action is different among different species [28]. Hence, to determine the function of IAA, future study will focus on the synergistic effect with other hormones on bolting in lettuce.

GAs is a large family of plant hormones that promote the relaxation of cell walls and cell elongation resulting in stem elongation, which plays an important role in the bolting of vegetables [29]. The three types of enzymes involved in the synthesis of biologically active GA from GA precursors are terpene synthases (TPSs), cytochrome P450 monooxygenases (P450s), and 2-oxoglutarate-dependent dioxygenases (2ODDs) [30]. In the current study, it was found that the GA-regulated protein 1-like (GRP1L) and 8 cytochrome P450 monooxygenases had increased abundance. Among them, P450s are key enzymes in the synthesis of GA, and they catalyze a number of oxidation steps in the middle part of the pathway [31]. However, needs to be the very specific P450s, and the identified P450s in this study were not the types involved in GA biosynthesis. The increased abundance of GA-regulated proteins indicated that the change of GA signal transduction might be consistent with the bolting. Researchers who found that exogenous GA caused a severe elongation of the lettuce stem [32], and promoted bolting in both a bolting-resistant lettuce line and a bolting-sensitive line [17]. GA could promote bud differentiation in the rape flower [33], the AtGA20ox1 could greatly contribute to internode and filament elongation in *Arabidopsis* [34], and the application of GA_3_ to spinach plants rapidly induced bolting [28]. T better determine the function of P450s in GA synthesis and its relationship with bolting, the type of P450s that are responsible for this needs to be determined, and a transgenosis analysis needs to be carried out in the future. Additionally, whether the GA signal transduction affects plant bolting may be a new research focus.

### 3.2. Proteins Related to Phosphorylation During Bolting in Lettuce Under a High Temperature

Protein phosphorylation is an important mechanism for the regulation of cellular responses to various external signals [35], and it regulates the basic processes of cell metabolism, such as cell division, differentiation, and growth development. In addition, protein phosphorylation plays an important role in hormone regulation as well as biological and abiotic stress responses [36]. According to the amino acid residue types of substrate proteins, protein kinases can be divided into the categories serine/threonine protein kinases (STPK), tyrosine protein kinases (TPK), and histidine protein kinases (HPK), tryptophan protein kinases and aspartate aminoacyl/glutamyl protein kinases. Among these, STPK is one of the most important protein kinases and regulates many cell life activities [37]. The receptor serine/threonine kinases (RSTK), which is a type of single transmembrane protein receptor, shows that STPK activity in the cell and always exerts a normal physiological function as a heterodimer. This protein kinase may primarily cause the phosphorylation of serine or threonine downstream signaling proteins, and it can pass extracellular signals into cells, and can achieve a variety of biological functions through its influence on gene transcription. Recently, many studies about the phosphorylated protein functions in some species have been reported, including peanuts [38], alfalfa [39], and *Arabidopsis* [40], have been reported. In this study, we found that four RSTKs in lettuce stems during bolting under a high temperature had decreased abundance, and one STPK had increased abundance, which affected the phosphorylation of serine or threonine in downstream signaling proteins, ans might further impact the bolting induced by high temperature.

### 3.3. Defense Proteins Play an Important Role in Defense Reaction and Hormone Metabolism During Bolting in Lettuce Under a High Temperature

Glutathione S-transferase (GST) is a common enzyme family found in bacteria, fungi, plants and animals, and has many biological functions. As a type of isozyme, GSTs are encoded by a large and complex gene family [41]. This kind of enzyme can catalyze the conjugate reaction of reduced glutathione and toxic, alien or oxidation products [42], protects cells from oxidative damage [43], and plays an important role in plant resistance to abiotic stresses, including chilling, drought, and high salt stress [44]. In this study, two of the GST family proteins, namely, glutathione *S*-transferase l3 (1.26) and glutathione *S*-transferase l3-like (1.22), were detected in bolting lettuce stems. Thus, GSTs are also found to be involved in the bolting process of lettuce under high temperature.

Ascorbic acid (AsA) is a common small-molecule antioxidant in higher plants that plays an important role in the resistance of plant cells to oxidative stress. AsA is an important and required cofactor of metabolic enzymes in the synthesis of secondary metabolites, such as ethylene, GA, and anthocyanin, and it also regulates cell growth. During the progress of bolting in *Arabidopsis*, the AsA content was greatly reduced in parallel with an increased expression of OgLEAFY, and the gene encoded a key transcription factor that integrates different flowering-inducing pathways [45]. In the current study, changes in the expression of AsA metabolism-related proteins likely lead to the increase of endogenous AsA content in lettuce stems, thereby indirectly promoting the synthesis of plant hormones, as well as initiating a protective function.

### 3.5. Proteins Associated with Photosynthesis During Bolting in Lettuce

Plant growth and development must be coordinated with metabolism, notably with the efficiency of photosynthesis and the uptake of nutrients. This coordination requires local connections between the hormone response and metabolic state, as well as long-distance connections between shoot and root tissues. In studies on Chinese cabbage [46], 19 expression sequence tags (ESTs) associated with bolting or flowering were isolated and cloned, and the blast results indicated that 15 of them were involved in the synthesis of anthocyanins, photosynthesis, and signal transduction. During the bolting process of Chinese cabbage, it was confirmed that photosynthesis and abiotic signal response genes, such as BrPIF4, BrPIF5, and BrCOLs, were highly expressed in the outer leaves [47]. In our study, the photosynthesis-related proteins have significant increased abundance in the bolting process under a high temperature, indicating that photosynthesis may be significantly enhanced. While our photosynthesis analysis was on the lettuce stem, these findings are similar to previous research on lettuce leaves. Some lettuce leaves will likely be injured under a high temperature, as a consequence of tissue necrosis, so the enhancement of stem photosynthesis might be a way to supplement affected leaf photosynthesis. This could be an explanation for the accumulation of a large amount of organic matter (sugar) in the stem, which is needed for the growth of the stem and bolting. The subsequent identification will be further confirmed by the determination of stem photosynthesis. Previous research shows that a relationship exists between photosynthesis and phytohormones in plants. Exogenous GA enhanced the expression of many key photosynthetic genes, such as GID1, RGA, GID2, and MYBGa, which is in agreement with the observed increase in the measurements of photosynthesis [48]. Huerta found that an extensive up-regulation of genes involved in photosynthesis and carbon utilization, and down-regulation of those involved in protein synthesis and ribosome biogenesis, were shown for the first time in plants with a higher GA content [49]. The maximum IAA content and ethylene evolution was noted when the upper leaves were removed, the photosynthetic rate and photosynthetic water-use efficiency showed a reverse trend, and the application of IAA could recover the photosynthesis and stomatal conductance of 50% of upper leaf removal plants [50]. Combining the changes in the hormone pathway, it was speculated that the increased abundance proteins involved in GA and IAA metabolism increased the content of endogenous GA and IAA, thereby enhancing photosynthesis and benefitting plant bolting. Comprehensively, it was suggested that the drastic changes in photosynthesis, carbon metabolism, ribosome biogenesis, glycolysis/gluconeogenesis, phenylpropanoid biosynthesis, tryptophan metabolism may play an important role in the inducement of bolting, and the enhanced function of photosynthesis and tryptophan metabolism may locate the important place. Reports have indicated that the tryptophan-dependent IAA synthesis pathway is an important route for IAA synthesis in plants [51]. Similarly, the elevated gene expression implicated in tryptophan metabolism matched the higher levels of IAA content observed in stems of plants subjected to high temperature (Figure 3).

### 3.6. Proteins Associated with Expansin During Bolting in Lettuce

Expansins were first discovered in cucumbers [52] Expansins are a class of cell wall proteins that uniquely induce pH-dependent cell wall elongation and alleviate wall pressure [53]. As an important part of plant cell wall, expandable protein makes cell wall component loose and cell stretch by means of enzyme catalysis [54]. Wittwer and Bukovac reported that the application of GA induced bolting and earlier flowering in lettuce [55]. Cosgrove found that gibberellin had similar effects on stem elongation and expansins [54]. In this study, we found that one expansin protein (expansin-like b1) had increased abundance in the lettuce stem during bolting induced by high temperature, which was significantly different from the control group. At the same time, a beta expansin precursor had decreased abundance. Therefore, expansin proteins might be related to bolting induced by high temperature in lettuce. Thus, we will focus on the relationship between expansin proteins and bolting in future study.

## 4. Materials and Methods

### 4.1. Plant Materials and Treatment

Seeds of GB-30 lettuce (*Lactuca sativa* L.), a variety that bolts easily, were numbered and conserved in our laboratory, sown in a sand/soil/peat (1:1:1 *v*/*v*) mixture, and grown in the Beijing University of Agriculture Experimental Station of Beijing under standard greenhouse conditions (14 h light; 300–1300 μmol/(m^2^ s); 20 ± 2 °C during the day; 13 ± 2 °C at night; 10 h dark; and 50–70% relative humidity). The seedlings were transplanted into 10 cm pots at the trefoil stage. When the lettuce plants developed the sixth true leaf, they were moved to a growth chamber under the following condition: Temperatures of 20/13 °C (day/night), a 14/10 h photoperiod, and 60% relative humidity for two days of acclimatization. After that, the plants were divided into two groups. The control group (group CK) was kept under the standard greenhouse conditions as described above. The other group (group H) was moved to another growth chamber and treated with high temperatures of 33 and 25 °C during the day and night, respectively. The other environmental conditions were unchanged. The stem lengths (in cm) of the control and treatment plants were measured every eight days using a ruler. At the same time, the blossom buds were observed by the stereoscopic microscope and paraffin methods [56] to define the progress of flower bud differentiation. After 32 days, stem samples from the control and treatment plants were collected, frozen in liquid nitrogen, and stored at −80 °C for further measurements on endogenous hormones and proteome analysis.

### 4.2. Endogenous Hormone Measurement

The endogenous hormone measurements were performed using enzyme-linked immune sorbent assays (ELISA), as previously described [57]. Standard auxin (IAA), gibberellin (GA_1+3_), zeatin (ZR), abscisic acid (ABA), Jasmonic acid methyl ester (JA-ME), and brassinolide (BR) (Sangon Biotech Co. Ltd., Shanghai, China) were used for calibration. The unit of endogenous hormone content was ng/g FW.

### 4.3. Exogenous Auxin (IAA) Treatment

After observing the effects of exogenous auxin at different concentrations (10, 40, 70, and 100 mg/L), 40 mg/L was chosen for the formal exogenous auxin treatment of bolting in the preparatory experiment. Plants at the sixth true leaf stage with uniform growth were selected and sprayed with 40 mg/L auxin. Water was used as a control. Twelve plants were used for each treatment, and the stem length (in cm) was measured using a ruler, every five days from the start of the treatment.

### 4.4. Protein Extraction

Approximately 2.5 g of each sample was ground into fine powder in liquid nitrogen. The powder was resuspended in 30 mL of 10% (*w*/*v*) trichloroacetic acid (TCA)/acetone (65 mM dithiothreitol (DTT)) in a 50 mL tube. The mixture was stored overnight (minimum duration) at −20 °C for precipitation. After centrifugation at 10,000 rpm for 30 min at 4 °C, the supernatant was discarded. Subsequently, 40 mL pre-cooling acetone was added and centrifuged at 7000 rpm for 15 min. This supernatant was also discarded and the pellet was washed three times with acetone. Afterwards, 200 uL lysis buffer (SDT buffer (4% (*v*/*v*) SDS, 100 mM Tris-HCl, 1 mM DTT, pH 7.6) was added to the precipitate, and placed on ice for 20 min after ultrasonic treatment for 30 min. After centrifugation at 12,000 rpm for 10 min at 4 °C, the supernatant was extracted. The precipitate was vacuum-dried. The total protein in the supernatant was quantified using the BCA Protein Assay Kit (Bio-Rad, Hercules, CA, USA).

### 4.5. Protein Digestion and iTRAQ Labeling

Protein digestion was performed according to the FASP procedure described by Wiśniewski and colleagues [58], and the resulting peptide mixture was labeled using the 8-plex iTRAQ reagent according to the manufacturer’s instructions (Applied Biosystems, Foster City, CA, USA). In brief, each sample of 200 μg of protein was incorporated into a 30 μL SDT buffer (4% (*v*/*v*) SDS, 100 mM DTT, 150 mM Tris-HCl pH 8.0). The detergent, DTT, and other low-molecular-weight components were removed using a UA buffer (8 M Urea, 150 mM Tris-HCl pH 8.0) and repeated ultrafiltration (Microcon units, 30 kD). Then, 100 μL 0.05 M iodoacetamide in UA buffer was added to block reduced cysteine residues, and the samples were incubated in the dark for 20 min. The filters were washed three times with 100 μL UA buffer and, subsequently, twice with 100 μL DS buffer (50 mM triethylammoniumbicarbonate at pH 8.5). Finally, the protein suspensions were digested at 37 °C overnight using 2 μg trypsin (Promega, Madison, WI, USA) in 40 μL DS buffer, and the resulting peptides were collected as a filtrate. The peptide content was estimated by UV light spectral density at 280 nm using an extinction coefficient of 1.1 of 0.1% (g/L) solution, which was calculated based on the frequency of tryptophan and tyrosine in vertebrate proteins. For labeling, each iTRAQ reagent was dissolved in 70 μL of ethanol and added to the respective peptide mixture. The experiment was performed in three independent biological replicates, and each independent biological replication consisted of a pool of three plants. The three independent biological replications of the control were labeled as (CK1)-113, (CK2)-114, and (CK3)-115, and the three independent biological replications of the treatment were labeled as (H1)-116, (H2)-117, and (H3)-118.

### 4.6. Peptide Fractionation with Strong Cation Exchange (SCX) Chromatography

The iTRAQ labeled peptides were fractionated by SCX chromatography using the AKTA Purifier system (GE Healthcare, Chicago, IL, USA). The dried peptide mixture was reconstituted and acidified with 2 mL buffer A (10 mM KH_2_PO_4_ in 25% (*v*/*v*) of ACN, pH 2.7) and loaded onto a PolySULFOETHYL 4.6 × 100 mm column (5 µm, 200 Å, PolyLC Inc, Columbia, MD, USA). The peptides were eluted with buffer B (500 mM KCl, 10 mM KH_2_PO_4_ in 25% (*v*/*v*) of ACN, pH 2.7) at a flow rate of 1 ml/min with the following gradients of 0–8% buffer B (500 mM KCl, 10 mM KH2PO4 in 25% of ACN, pH 3.0) for 22 min, 8–52% buffer B from 22–47 min, 52–100% buffer B from 47–50 min, 100% buffer B from 50–58 min, and then, after 58 min, buffer B was reset to 0%. The elution was monitored by absorbance at 214 nm, and fractions were collected every minute. The collected fractions were combined into 15 fractions and desalted on C18 Cartridges (Empore™ SPE Cartridges C18 (standard density), bed I.D 7 mm, volume 3 mL, Sigma, St. Louis, MI, USA). Each fraction was concentrated by vacuum centrifugation and reconstituted in 40 µL of 0.1% (*v*/*v*) acetic acid. All samples were stored at −80 °C until LC-MS/MS analysis.

### 4.7. Liquid Chromatography (LC)-Electrospray Ionization (ESI) Tandem MS (MS/MS) Analysis

Experiments were performed on a Q-Exactive mass spectrometer coupled with Easy nLC (Proxeon Biosystems, now Thermo Fisher Scientific). Into each fraction, 10 μL was injected for nano LC-MS/MS analysis. The peptide mixture (1–2 μg) was loaded onto a C18–reversed phase column (Thermo Scientific Easy Column, 10 cm long, 75 μm inner diameter, 3 μm resin) in buffer A (0.1% (*v*/*v*) formic acid) and separated with a linear gradient of buffer B (80% (*v*/*v*) acetonitrile and 0.1% (*v*/*v*) Formic acid) at a flow rate of 250 nL/min, controlled by IntelliFlow technology for 140 min. MS data were acquired using a data-dependent top 10 method, which dynamically chooses the most abundant precursor ions from the survey scan (300–1800 *m*/*z*) for HCD fragmentation. The determination of the target value is based on predictive Automatic Gain Control (pAGC). The dynamic exclusion duration was 60 s. Survey scans were acquired at a resolution of 70,000 at *m*/*z* 200, and the resolution for HCD spectra was set to 17,500 at *m*/*z* 200. Normalized collision energy was 30 eV, and the underfill ratio, which specifies the minimum percentage of the target value likely to be reached at the maximum fill time, was defined as 0.1%. The instrument was run with peptide recognition mode enabled.

### 4.8. Database Search and Protein Quantification

MS/MS spectra were searched using the MASCOT engine (Matrix Science, London, UK; version 2.2) embedded into Proteome Discoverer 1.3 (Thermo Electron, San Jose, CA, USA), against Lactuca.Unigene.pep.fasta (lettuce protein database translated from transcriptome, created by our laboratory). For protein identification, the following options were used: Peptide mass tolerance = 20 ppm, MS/MS tolerance = 0.1 Da, Enzyme = Trypsin, Max Missed cleavage = 2, Fixed modification: Carbamidomethyl (C), iTRAQ 8-plex (K), iTRAQ 8-plex (N-term), Variable modification: Oxidation (M), iTRAQ8plex (Y). Each confident protein identification and quantification required at least one unique peptide, and the proteins with one unique peptide were supplied high quality spectra in Table S3. The false discovery rate (FDR) of identified proteins was ≤0.01.

The relative quantification of proteins was based on the strength of the reporter ion, which reflects the relative abundance of peptides. The fold-change was obtained according to different comparison groups (control and high-temperature treatment), through the reporter ion ratio labeled with different isotopes, as described above. In the identified proteins, the fold-changes of >1.20 or <0.84, and the *p*-values of <0.05 using one sample t test, were considered as significant.

### 4.9. Bioinformatics Analysis of Proteins

Functional category analysis was performed with Blast2GO software (http://www.geneontology.org) [59]. The online Kyoto Encyclopedia of Genes and Genomes (KEGG) database (http://www.genome.jp/kegg/) was used to retrieve their KEGG Orthology (KO) and the data were subsequently mapped on pathways in KEGG [60]. The corresponding KEGG pathways were extracted. To further explore the impact of proteins with differential abundance in the cell physiological processes of cells and discover internal relations between proteins with differential abundance, enrichment analysis was performed. GO enrichment on three ontologies (biological process, molecular function, and cellular component), and KEGG pathway enrichment analyses were applied based on the Fisher’s exact test, considering the whole quantified protein annotations as the background dataset. The Benjamini–Hochberg correction for multiple testing was further applied to adjust the derived *p*-values. Only functional categories and pathways with *p*-values <0.05 were considered as significant. The studied protein-relative abundance data were used to perform hierarchical clustering analysis. For this purpose, Cluster 3.0 (http://bonsai.hgc.jp/~mdehoon/software/cluster/software.htm) and the Java Treeview software (http://jtreeview.sourceforge.net) were used. The Euclidean distance algorithm for similarity measurement and the average linkage clustering algorithm for clustering were selected when performing hierarchical clustering.

The workflow in the proteome is shown in Figure S1.

### 4.10. Total RNA Extraction and Real-Time PCR

The total RNA was extracted using the RNA pure Total RNA Kit (Aidlab Biotech, Beijing, China) according to the manufacturer’s instructions. The RNA samples were reversely transcribed into cDNAs using TransScript First-Strand cDNA Synthesis SuperMix (TransGen Biotech, Beijing, China). The procedure was as follows: RNA (2 µg) mixed with 1 μL Oligod (T) 18 (0.5 μg/ μL), 2 × TS Reaction Mix (10 µL) and, TransScript RT/RI Enzyme Mix (1 µL) with an additional 20 µL of RNase-free Water to. The mixture was mixed gently and incubated at 42 °C for 15 min. The reaction was terminated by incubation at 85 °C for 5 s, and the cDNAs of the product were stored at −20 °C. The cDNA samples were used as a template, then mixed with 200 nmol primer and SYBR Green PCR Real Master Mix (Takara, Kusatsu, Japan) for real-time PCR analysis using Bio-Rad CFX 96 real-time PCR instruments and CFX manager software ver 3.0 (Bio-Rad laboratories, California, USA). The temperature procedure was as follows: 94 °C for 3 min, 32 cycles of 94 °C for 30 s, 57 °C for 30 s, and 72 °C for 20 s. The fluorescence signal was collected during the elongation of every cycle at 72 °C. The 18S was used as an internal standard for normalization. The primers used in qRT-PCR are listed in Table S5.

### 4.11. Statistical Analysis

All tests were performed in three replicates. For the measurement of stem length, each biological replicate had nine samples from nine plants. For the observation of flower bud differentiation, each biological replicate had five samples from five plants. For physiology and proteome analyses, three different stems were pooled together as one biological sample, and this was done three times to produce three independent biological replicates (of three pooled stems) for both physiology and proteome analysis. The presented data represent the means ± SD of three replications, and were statistically analyzed using an analysis of variance (ANOVA) by SPSS 10.0 (International Business Machine, Chicago, IL, USA). Tukey’s test was used to identify significant differences among groups (*p* < 0.05, *p* < 0.01). Figures representing the physiological parameters were drawn using Origin Pro 8.0 SR4 (Origin Lab, Northampton, MA, USA) and Microsoft Office PowerPoint 2007. Western blot immunoreactive protein bands were quantified by densitometry using ImageLab 3.0 software (Bio-Rad, Hercules, CA, USA).

## 5. Conclusions

The bolting of plants is usually induced by a wide variety of biological changes. Here, we observed the phenotypic changes of lettuce bolting induced by a high temperature of 33 °C, which are in sharp contrast to lettuce subjected to the normal growth temperature of 20 °C, under which condition no bolting occurred. The activity of enzymes involved in protein synthesis and defense systems was functionally enhanced. Subsequently, the functional classes associated with reproductive growth, such as hormone metabolism, were highly activated, especially for IAA. These findings by proteomics were in agreement with the validation in the physiology and gene expression of IAA metabolism. Furthermore, the functions of IAA may promote photosynthesis. Thus, the synergistic reactions of these metabolisms led to the synthesis of expansin and cell cycle proteins, eventually resulting in plant bolting.

## Supplementary Materials

The supplementary materials are available online at http://www.mdpi.com/1422-0067/19/10/2967/s1.
