# Supplementary material for: Quantitative Proteomics Analysis of Lettuce (Lactuca sativa L.) Reveals Molecular Basis-Associated Auxin and Photosynthesis with Bolting Induced by High Temperature
Source: Int J Mol Sci. 2018 Sep 28;19(10):2967. doi: 10.3390/ijms19102967 (PMC6213495; doi:10.3390/ijms19102967)
Supplement: Supplementary file 1 [file ijms-19-02967-s001.zip › ijms-336629 Supplementary/supplemental Table S2.docx]

Supplemental Table S2 The proteins changed significantly in abundance in lettuce stem after high temperature treatment

| NO. | Accession no. | Protein name | Coverage (%)^a^ | Fold change (treatment  /control)^b^ | P-value |
| --- | --- | --- | --- | --- | --- |
|  | c38769.graph_c0 | photosystem i reaction center subunit chloroplastic | 5.17 | 1.64 | 0.000005 |
|  | c39429.graph_c0 | rossmann-like alpha beta alpha sandwich fold | 34.04 | 1.22 | 0.000009 |
|  | c38735.graph_c0 | lipid transfer protein | 43.59 | 1.23 | 0.000015 |
|  | c39341.graph_c0 | nadp-dependent glyceraldehyde-3-phosphate dehydrogenase | 42.34 | 1.26 | 0.000034 |
|  | c33137.graph_c0 | aspartic proteinase asp1 | 14.11 | 1.21 | 0.000054 |
|  | c40606.graph_c0 | six-bladed beta- -like protein | 6.65 | 1.35 | 0.000070 |
|  | c42400.graph_c0 | annexin d2-like | 37.97 | 1.61 | 0.000087 |
|  | c27269.graph_c0 | beta partial | 82.08 | 1.21 | 0.000098 |
|  | c40109.graph_c0 | ferritin- chloroplastic-like | 49.41 | 1.49 | 0.000118 |
|  | c37054.graph_c0 | protein argonaute 10 | 8.35 | 1.26 | 0.000137 |
|  | c24628.graph_c0 | phospho-2-dehydro-3-deoxyheptonate aldolase chloroplastic-like | 26.19 | 1.42 | 0.000153 |
|  | c38759.graph_c0 | photosystem i reaction center subunit chloroplastic-like | 23.08 | 1.82 | 0.000207 |
|  | c38966.graph_c0 | 50s ribosomal protein chloroplastic | 14.14 | 1.44 | 0.000218 |
|  | c38762.graph_c0 | chlorophyll a-b binding protein chloroplastic | 28.1 | 1.86 | 0.000247 |
|  | c15919.graph_c0 | caffeic acid o-methyltransferase | 49.86 | 1.25 | 0.000293 |
|  | c41814.graph_c0 | beta-hydroxyisobutyryl- hydrolase 1 | 16.93 | 1.21 | 0.000295 |
|  | c42266.graph_c0 | germin-like protein 2-1 | 25.58 | 1.43 | 0.000311 |
|  | c24976.graph_c0 | cytochrome p450 | 32.67 | 1.35 | 0.000334 |
|  | c39100.graph_c0 | bet v i domain-containing protein | 58.94 | 1.33 | 0.000350 |
|  | c37554.graph_c0 | amidase family protein | 28.66 | 1.58 | 0.000354 |
|  | c38193.graph_c0 | esterase isoform x1 | 44.1 | 1.61 | 0.000363 |
|  | c36574.graph_c0 | chlorophyll a-b binding protein chloroplastic-like | 35.54 | 1.43 | 0.000366 |
|  | c35879.graph_c0 | chlorophyll a-b binding protein chloroplastic-like | 32.45 | 1.52 | 0.000370 |
|  | c36810.graph_c1 | udp-glycosyltransferase 76g1 | 7.43 | 1.42 | 0.000374 |
|  | c23945.graph_c0 | polyphenol oxidase precursor | 40.9 | 1.57 | 0.000399 |
|  | c16356.graph_c0 | spermidine synthase | 16.84 | 1.30 | 0.000445 |
|  | c32741.graph_c0 | epoxide hydrolase | 9.55 | 1.45 | 0.000456 |
|  | c17368.graph_c0 | hypothetical protein Ccrd_011733 | 13.46 | 3.22 | 0.000458 |
|  | c39525.graph_c0 | probable nucleoredoxin 1 | 32.69 | 1.65 | 0.000467 |
|  | c37455.graph_c0 | sulfotransferase domain-containing protein | 3.98 | 2.03 | 0.000474 |
|  | c24261.graph_c0 | annexin d3 | 31.55 | 1.25 | 0.000487 |
|  | c41527.graph_c0 | bisphosphoglycerate-independent phosphoglycerate mutase | 15 | 1.41 | 0.000496 |
|  | c22513.graph_c0 | outer envelope protein chloroplastic | 10.34 | 1.24 | 0.000500 |
|  | c36071.graph_c1 | beta-glucosidase 12-like | 11.65 | 1.75 | 0.000502 |
|  | c39250.graph_c0 | upf0603 protein chloroplastic | 17.01 | 2.09 | 0.000527 |
|  | c41306.graph_c0 | endoplasmin homolog | 43.47 | 1.29 | 0.000545 |
|  | c39269.graph_c0 | mannose-binding lectin | 39.46 | 2.32 | 0.000578 |
|  | c42680.graph_c0 | heat shock protein 83 | 40.34 | 2.24 | 0.000586 |
|  | c38215.graph_c0 | germacrene a synthase | 40.75 | 1.56 | 0.000646 |
|  | c45667.graph_c0 | cingulin-like protein 1 | 32.13 | 1.30 | 0.000653 |
|  | c16709.graph_c0 | fructan 1-exohydrolase iib | 36.14 | 1.55 | 0.000664 |
|  | c39580.graph_c0 | sec14 cytosolic factor family protein phosphoglyceride transfer family protein isoform 2 | 73.96 | 1.54 | 0.000694 |
|  | c38799.graph_c0 | plasma membrane intrinsic protein partial | 18.27 | 1.23 | 0.000697 |
|  | c22141.graph_c0 | endonuclease 2 | 3.87 | 1.88 | 0.000740 |
|  | c32791.graph_c0 | elongation factor 1-alpha | 31.86 | 1.27 | 0.000770 |
|  | c41444.graph_c0 | gibberellin-regulated protein 1-like | 8.08 | 2.18 | 0.000798 |
|  | c41649.graph_c0 | early nodulin-like protein 1 | 14.68 | 1.39 | 0.000812 |
|  | c37424.graph_c1 | plant intracellular ras-group-related lrr protein 4-like | 7.19 | 1.30 | 0.000814 |
|  | c40971.graph_c0 | chromatin structure-remodeling complex subunit rsc1-like isoform x1 | 12.04 | 1.37 | 0.000851 |
|  | c16202.graph_c0 | chlorophyll a-b binding protein chloroplastic | 13.06 | 1.82 | 0.000908 |
|  | c25377.graph_c0 | 23 kda jasmonate-induced | 38.05 | 2.38 | 0.000911 |
|  | c23035.graph_c0 | isoflavone reductase-like protein | 40.48 | 1.32 | 0.000919 |
|  | c39326.graph_c0 | 50s ribosomal protein chloroplastic | 10.98 | 1.31 | 0.000921 |
|  | c29954.graph_c0 | oxygen-evolving enhancer protein chloroplastic | 62.61 | 1.61 | 0.000926 |
|  | c39224.graph_c0 | non-symbiotic hemoglobin 2 | 25.47 | 1.44 | 0.000928 |
|  | c24935.graph_c1 | phox bem1p | 33.33 | 1.28 | 0.000978 |
|  | c32587.graph_c0 | ribulose- -bisphosphate carboxylase oxygenase small subunit | 45.2 | 1.68 | 0.001023 |
|  | c38925.graph_c0 | polyphenol oxidase precursor | 13.44 | 2.15 | 0.001053 |
|  | c28864.graph_c0 | probable linoleate 9s-lipoxygenase 5 | 2.98 | 1.23 | 0.001111 |
|  | c15385.graph_c0 | calcineurin subunit b | 4.44 | 1.36 | 0.001125 |
|  | c40675.graph_c0 | selenocysteine methyltransferase family protein | 38.46 | 1.58 | 0.001131 |
|  | c17189.graph_c0 | carotenoid cleavage dioxygenase 1 | 13.68 | 1.22 | 0.001140 |
|  | c40505.graph_c0 | kda class i heat shock protein | 23.08 | 1.44 | 0.001143 |
|  | c30451.graph_c0 | tubulin beta-1 chain | 64.29 | 1.63 | 0.001150 |
|  | c40472.graph_c0 | nadh dehydrogenase | 14.37 | 1.36 | 0.001150 |
|  | c34798.graph_c1 | PREDICTED: miraculin-like | 18.78 | 1.67 | 0.001185 |
|  | c41596.graph_c0 | hydroquinone glucosyltransferase-like | 10.99 | 1.53 | 0.001196 |
|  | c38750.graph_c0 | chlorophyll a-b binding protein chloroplastic | 24.31 | 1.57 | 0.001213 |
|  | c38054.graph_c1 | secoisolariciresinol dehydrogenase-like | 51.29 | 1.38 | 0.001226 |
|  | c38677.graph_c0 | sucrose synthase | 55.16 | 1.27 | 0.001234 |
|  | c42870.graph_c0 | peroxidase 12-like | 7 | 1.41 | 0.001238 |
|  | c36881.graph_c0 | snare-interacting protein keule | 17.37 | 1.21 | 0.001255 |
|  | c28510.graph_c1 | cytochrome p450 cyp72a219-like | 16.13 | 1.20 | 0.001256 |
|  | c38978.graph_c0 | polyadenylate-binding protein-interacting protein 2-like | 14.48 | 1.60 | 0.001258 |
|  | c30451.graph_c1 | beta- partial | 61.74 | 1.20 | 0.001259 |
|  | c39225.graph_c0 | plasma membrane-associated cation-binding protein 1 | 19.6 | 1.39 | 0.001264 |
|  | c36600.graph_c0 | bet v i domain-containing protein | 14.67 | 1.84 | 0.001274 |
|  | c39776.graph_c0 | caffeoylshikimate esterase-like | 36.73 | 1.31 | 0.001292 |
|  | c15980.graph_c0 | nucleotide- alpha-beta plait | 62.35 | 1.35 | 0.001303 |
|  | c32705.graph_c0 | kda class i heat shock | 33.55 | 1.59 | 0.001307 |
|  | c34629.graph_c0 | chaperonin cpn60- mitochondrial | 39.47 | 1.29 | 0.001329 |
|  | c37923.graph_c1 | trihelix transcription factor gt-2-like | 7.45 | 1.53 | 0.001384 |
|  | c41022.graph_c0 | early nodulin-like protein 1 | 25.25 | 1.26 | 0.001405 |
|  | c36923.graph_c0 | mannose-binding lectin | 35.57 | 1.31 | 0.001435 |
|  | c28221.graph_c0 | protein of unknown function DUF2296 | 33.33 | 1.21 | 0.001440 |
|  | c39059.graph_c0 | thylakoid lumenal 19 kda chloroplastic | 5.76 | 1.75 | 0.001454 |
|  | c39396.graph_c0 | barwin-like endoglucanase | 3.95 | 1.61 | 0.001488 |
|  | c24171.graph_c0 | fam10 family protein at4g22670 | 17.78 | 1.26 | 0.001503 |
|  | c41928.graph_c0 | germin-like protein subfamily 1 member 1 | 26.15 | 1.26 | 0.001504 |
|  | c42673.graph_c0 | hsp70-hsp90 organizing protein 3-like | 63.64 | 1.29 | 0.001511 |
|  | c38928.graph_c0 | heat shock protein cysteine-rich domain-containing protein | 9.59 | 1.85 | 0.001593 |
|  | c30216.graph_c0 | acetyl-CoA c-acetyltransferase 3 | 52.94 | 1.21 | 0.001633 |
|  | c29484.graph_c0 | bet v i domain-containing protein | 67.19 | 1.50 | 0.001633 |
|  | c17953.graph_c0 | sulfotransferase domain-containing protein | 11.73 | 1.85 | 0.001698 |
|  | c17713.graph_c0 | uncharacterized protein | 27.64 | 1.72 | 0.001742 |
|  | c32426.graph_c0 | protein exordium-like 2 | 5.84 | 1.69 | 0.001774 |
|  | c42601.graph_c0 | bet v i domain-containing protein | 29.69 | 2.51 | 0.001792 |
|  | c17715.graph_c0 | fasciclin-like arabinogalactan protein 11 | 12.25 | 1.81 | 0.001797 |
|  | c17957.graph_c0 | monocopper oxidase-like protein sku5 | 22.71 | 1.31 | 0.001798 |
|  | c16555.graph_c1 | actin | 60.79 | 1.57 | 0.001834 |
|  | c16105.graph_c0 | PREDICTED: uncharacterized protein LOC101222618 | 49.25 | 1.42 | 0.001837 |
|  | c32457.graph_c0 | biogenesis of lysosome-related organelles complex 1 subunit 2 | 20 | 1.25 | 0.001863 |
|  | c27213.graph_c0 | calmodulin-binding partial | 2.14 | 1.54 | 0.001866 |
|  | c40522.graph_c0 | germacrene a oxidase | 24.59 | 1.62 | 0.001886 |
|  | c38439.graph_c0 | lipase plat lh2 | 37.67 | 1.44 | 0.001959 |
|  | c40925.graph_c0 | thylakoid lumenal protein chloroplastic | 5.15 | 1.49 | 0.001988 |
|  | c41597.graph_c0 | hypothetical protein Ccrd_010466 | 81.63 | 1.47 | 0.002009 |
|  | c35526.graph_c0 | protein catalytic domain-containing protein | 5.34 | 1.58 | 0.002029 |
|  | c25171.graph_c0 | cysteine proteinase inhibitor a-like | 22.45 | 1.32 | 0.002082 |
|  | c19150.graph_c0 | tumor-related protein | 12.75 | 2.22 | 0.002097 |
|  | c16401.graph_c0 | 1-phosphatidylinositol phosphodiesterase-related family protein | 10.69 | 1.38 | 0.002174 |
|  | c37259.graph_c0 | intracellular protein transport protein uso1-like | 6.93 | 1.76 | 0.002183 |
|  | c20986.graph_c0 | dsba oxidoreductase family protein isoform 2 | 14.35 | 1.24 | 0.002184 |
|  | c36917.graph_c0 | caffeate o-methyltransferase family | 53.82 | 1.41 | 0.002204 |
|  | c39834.graph_c0 | protein thylakoid chloroplastic | 5.98 | 1.39 | 0.002207 |
|  | c25152.graph_c0 | tpa: membrane h(+)-atpase1 | 52.14 | 1.35 | 0.002218 |
|  | c15792.graph_c0 | sulfotransferase domain-containing protein | 20.55 | 1.54 | 0.002222 |
|  | c17618.graph_c0 | pectinesterase 3 | 25.29 | 1.37 | 0.002224 |
|  | c39243.graph_c0 | 50s ribosomal protein chloroplastic | 7.6 | 1.34 | 0.002232 |
|  | c23010.graph_c1 | protochlorophyllide reductase | 25.06 | 1.39 | 0.002263 |
|  | c39172.graph_c0 | remorin 1 | 54.26 | 1.41 | 0.002282 |
|  | c39285.graph_c0 | abc transporter b family member 19 | 15.65 | 1.22 | 0.002287 |
|  | c33816.graph_c0 | nad-dependent malic enzyme 59 kda mitochondrial | 52.94 | 1.32 | 0.002305 |
|  | c41990.graph_c0 | rna-binding protein lupus la | 2.5 | 1.23 | 0.002316 |
|  | c29485.graph_c0 | small edrk-rich factor 2 | 37.31 | 1.69 | 0.002385 |
|  | c35935.graph_c0 | l-ascorbate peroxidase peroxisomal | 21.13 | 1.22 | 0.002401 |
|  | c16079.graph_c0 | light-induced chloroplastic | 25.55 | 1.22 | 0.002408 |
|  | c40527.graph_c0 | alpha-soluble nsf attachment | 52.6 | 1.20 | 0.002415 |
|  | c41999.graph_c0 | isoform 1 | 42.21 | 1.38 | 0.002424 |
|  | c39293.graph_c0 | protein exordium-like 2 | 32.34 | 1.51 | 0.002433 |
|  | c38751.graph_c0 | photosystem i reaction center subunit chloroplastic-like | 10.2 | 1.78 | 0.002450 |
|  | c21729.graph_c0 | uncharacterized protein | 10.33 | 1.28 | 0.002451 |
|  | c39090.graph_c0 | lim domain-containing protein wlim1 | 8.65 | 1.56 | 0.002486 |
|  | c38953.graph_c0 | photosystem i reaction center subunit iv chloroplastic | 29.63 | 1.41 | 0.002493 |
|  | c23788.graph_c1 | uncharacterized protein | 50 | 1.28 | 0.002523 |
|  | c39005.graph_c0 | ribulose bisphosphate carboxylase oxygenase chloroplastic | 33.9 | 1.61 | 0.002537 |
|  | c29625.graph_c0 | serine arginine-rich splicing factor rs2z33-like isoform x2 | 2.36 | 1.78 | 0.002540 |
|  | c32285.graph_c0 | phosphoribulokinase family protein | 26.17 | 1.36 | 0.002556 |
|  | c41118.graph_c0 | plant intracellular ras-group-related lrr protein 3-like | 20 | 1.32 | 0.002614 |
|  | c38743.graph_c0 | oxygen-evolving enhancer protein 3- chloroplastic | 37.5 | 1.89 | 0.002635 |
|  | c39091.graph_c0 | atp synthase gamma chloroplastic | 4.81 | 1.55 | 0.002664 |
|  | c25310.graph_c0 | uncharacterized protein | 63.64 | 1.60 | 0.002703 |
|  | c40449.graph_c0 | nad -binding rossmann-fold superfamily protein | 3.73 | 1.28 | 0.002715 |
|  | c38865.graph_c0 | copper chaperone | 55.26 | 1.26 | 0.002715 |
|  | c40257.graph_c0 | 23 kda jasmonate-induced | 53.92 | 2.11 | 0.002747 |
|  | c36677.graph_c0 | chloramphenicol acetyltransferase-like domain-containing protein | 25.77 | 1.27 | 0.002783 |
|  | c24201.graph_c0 | uncharacterized protein | 14 | 1.39 | 0.002817 |
|  | c43185.graph_c0 | udp-glycosyltransferase 91a1-like | 3.02 | 1.32 | 0.002837 |
|  | c17892.graph_c0 | protein iq-domain 1-like | 13.11 | 1.25 | 0.002849 |
|  | c39357.graph_c0 | rhodanese-like domain-containing protein chloroplastic | 20.65 | 1.47 | 0.002850 |
|  | c39498.graph_c0 | photosystem ii stability assembly factor chloroplastic | 3.13 | 1.57 | 0.002887 |
|  | c25039.graph_c0 | probable polygalacturonase isoform x1 | 23.03 | 1.21 | 0.002908 |
|  | c38767.graph_c0 | unnamed protein product | 72.38 | 1.51 | 0.002917 |
|  | c39558.graph_c0 | glycerophosphoryl diester phosphodiesterase | 15.96 | 1.21 | 0.002926 |
|  | c40158.graph_c0 | mannose-binding lectin | 12.24 | 1.35 | 0.002927 |
|  | c16861.graph_c0 | bifunctional epoxide hydrolase 2-like | 21.27 | 1.52 | 0.002928 |
|  | c17730.graph_c0 | dessication responsive protein | 36.36 | 1.30 | 0.002962 |
|  | c30271.graph_c0 | kunitz type trypsin inhibitor | 35.48 | 1.32 | 0.002964 |
|  | c15636.graph_c0 | phytanoyl- dioxygenase | 9.19 | 1.29 | 0.002970 |
|  | c38281.graph_c0 | linoleate 13s-lipoxygenase 2- chloroplastic-like | 3.55 | 1.85 | 0.003002 |
|  | c39350.graph_c0 | ferritin- chloroplastic-like | 23.7 | 1.65 | 0.003003 |
|  | c40007.graph_c0 | mlp-like protein 423 | 72.37 | 1.60 | 0.003056 |
|  | c16612.graph_c0 | kda class i heat shock | 13.01 | 1.56 | 0.003058 |
|  | c22978.graph_c0 | electron transport accessory domain containing protein | 21.48 | 2.04 | 0.003097 |
|  | c38296.graph_c1 | glucose ribitol dehydrogenase | 18.15 | 1.23 | 0.003137 |
|  | c35972.graph_c1 | filament-like plant protein 7 isoform x2 | 8.27 | 1.29 | 0.003152 |
|  | c33282.graph_c0 | interactor of constitutive active rops 3-like | 3.9 | 1.25 | 0.003158 |
|  | c38832.graph_c0 | xyloglucan endotransglucosylase hydrolase | 30.98 | 1.89 | 0.003160 |
|  | c40242.graph_c0 | double-stranded dna-binding family protein | 24.81 | 1.32 | 0.003172 |
|  | c32922.graph_c0 | eg2771 | 18.23 | 1.35 | 0.003193 |
|  | c16835.graph_c0 | thylakoid lumenal 29 kda chloroplastic | 34.57 | 1.25 | 0.003204 |
|  | c44887.graph_c0 | random slug protein 5-like | 20 | 1.46 | 0.003216 |
|  | c17618.graph_c1 | pectin methylesterase 3 | 8.68 | 1.40 | 0.003232 |
|  | c38927.graph_c0 | 30s ribosomal protein chloroplastic | 16.67 | 1.40 | 0.003304 |
|  | c38822.graph_c0 | chlorophyll a-b binding protein chloroplastic | 5.28 | 1.71 | 0.003309 |
|  | c24743.graph_c0 | vesicle-associated protein 1-3 | 11.17 | 1.23 | 0.003317 |
|  | c41694.graph_c0 | thaumatin-like protein | 30.08 | 1.36 | 0.003343 |
|  | c40178.graph_c0 | thioredoxin chloroplastic | 15.63 | 1.42 | 0.003356 |
|  | c41575.graph_c0 | glucose ribitol dehydrogenase | 34.14 | 1.27 | 0.003371 |
|  | c16263.graph_c0 | plastid 3-keto-acyl-acp synthase i | 7.64 | 1.37 | 0.003489 |
|  | c49109.graph_c0 | heat shock cognate 71 kda | 39.76 | 1.75 | 0.003492 |
|  | c33136.graph_c0 | protein dj-1 homolog d | 14.5 | 1.27 | 0.003519 |
|  | c29829.graph_c0 | protein of unknown function DUF4057 | 43.42 | 1.47 | 0.003548 |
|  | c25227.graph_c2 | ras-related protein rabb1c | 45.45 | 1.22 | 0.003636 |
|  | c16447.graph_c0 | dnaj homolog mitochondrial-like | 13.43 | 1.24 | 0.003673 |
|  | c39051.graph_c0 | ras-related protein rab7 | 75.73 | 1.21 | 0.003706 |
|  | c31639.graph_c0 | cytochrome p450 93a3-like | 5.99 | 1.28 | 0.003721 |
|  | c17993.graph_c0 | ref srpp-like protein at1g67360 | 30.31 | 1.51 | 0.003723 |
|  | c32626.graph_c0 | photosystem i reaction center subunit chloroplastic-like | 22.31 | 1.58 | 0.003828 |
|  | c49262.graph_c0 | acyl-protein thioesterase 2-like | 5.33 | 1.25 | 0.003868 |
|  | c42112.graph_c0 | serine arginine-rich splicing factor sc35 | 24 | 1.25 | 0.003959 |
|  | c40185.graph_c0 | lachrymatory-factor synthase-like | 43.18 | 1.32 | 0.003982 |
|  | c40698.graph_c0 | desiccation protectant protein lea14 homolog | 7.95 | 1.62 | 0.003984 |
|  | c31954.graph_c1 | protein atp binding site-containing protein | 12.83 | 1.30 | 0.004021 |
|  | c39173.graph_c0 | upstream activation factor subunit spp27-like | 4.67 | 1.22 | 0.004030 |
|  | c34919.graph_c0 | tata-binding protein-associated factor 2n-like | 25.22 | 1.45 | 0.004031 |
|  | c39753.graph_c0 | cytochrome p450 71a22-like | 36.22 | 1.24 | 0.004036 |
|  | c39020.graph_c0 | thioredoxin | 27.72 | 1.26 | 0.004044 |
|  | c39117.graph_c0 | uncharacterized protein | 5.97 | 1.37 | 0.004048 |
|  | c29717.graph_c0 | uncharacterized protein | 49.11 | 1.79 | 0.004062 |
|  | c41323.graph_c0 | deoxynucleoside kinase | 9.86 | 1.51 | 0.004073 |
|  | c38761.graph_c0 | photosystem i reaction center subunit chloroplastic | 7.04 | 2.01 | 0.004121 |
|  | c15954.graph_c0 | atp synthase cf1 beta subunit | 57.8 | 1.30 | 0.004122 |
|  | c37349.graph_c0 | alcohol c-terminal | 34.24 | 1.28 | 0.004151 |
|  | c15490.graph_c0 | aspartate cytoplasmic | 18.86 | 1.38 | 0.004174 |
|  | c39120.graph_c0 | early nodulin-like protein 2 | 23.98 | 1.40 | 0.004179 |
|  | c15959.graph_c0 | catalase | 54.18 | 1.35 | 0.004190 |
|  | c40168.graph_c0 | probable calcium-binding protein cml20 | 26.79 | 1.22 | 0.004194 |
|  | c40223.graph_c0 | xylose isomerase | 46.47 | 1.25 | 0.004199 |
|  | c29667.graph_c0 | cytidine deaminase 1 | 6.35 | 1.20 | 0.004211 |
|  | c38746.graph_c0 | rubisco activase isoform 2 | 24.61 | 1.39 | 0.004222 |
|  | c43028.graph_c0 | ankyrin repeat family protein | 24.78 | 1.22 | 0.004318 |
|  | c19782.graph_c0 | cytochrome p450 | 15.87 | 1.37 | 0.004319 |
|  | c15968.graph_c0 | aldolase-type tim barrel family protein isoform 1 | 18.97 | 1.31 | 0.004324 |
|  | c39079.graph_c0 | tetraspanin-8-like | 23.42 | 1.39 | 0.004454 |
|  | c42292.graph_c0 | cysteine-rich repeat secretory protein 12 | 10.24 | 2.08 | 0.004524 |
|  | c40544.graph_c0 | kirola-like | 12.42 | 1.70 | 0.004573 |
|  | c34529.graph_c0 | Conserved hypothetical protein CHP01589, | 9.57 | 1.38 | 0.004587 |
|  | c25237.graph_c0 | probable glycerophosphoryl diester phosphodiesterase 2 | 12.37 | 1.27 | 0.004622 |
|  | c42458.graph_c0 | 3-hydroxyisobutyryl- hydrolase-like protein mitochondrial isoform x1 | 4.35 | 1.23 | 0.004656 |
|  | c38856.graph_c0 | 30s ribosomal protein chloroplastic | 40.22 | 1.21 | 0.004665 |
|  | c39244.graph_c0 | 50s ribosomal protein chloroplastic | 5.42 | 1.49 | 0.004698 |
|  | c34364.graph_c0 | grip and coiled-coil domain-containing protein 2 isoform x5 | 2.88 | 1.34 | 0.004716 |
|  | c32245.graph_c0 | transcription factor myb1r1 | 6.72 | 1.33 | 0.004721 |
|  | c36125.graph_c1 | probable aldo-keto reductase 2 | 29.33 | 1.20 | 0.004741 |
|  | c23711.graph_c0 | probable dna double-strand break repair rad50 atpase | 20.57 | 1.38 | 0.004836 |
|  | c32582.graph_c0 | protein curvature thylakoid chloroplastic | 18.63 | 1.43 | 0.004847 |
|  | c33588.graph_c1 | math domain-containing protein at5g43560-like | 4.52 | 1.22 | 0.004906 |
|  | c34927.graph_c0 | isoform 1 | 1.2 | 1.28 | 0.004911 |
|  | c17525.graph_c0 | cytochrome p450 | 16.7 | 1.43 | 0.004926 |
|  | c38731.graph_c0 | 2fe-2s ferredoxin superfamily protein | 6.45 | 2.12 | 0.004941 |
|  | c42085.graph_c0 | calcyclin-binding protein | 9.77 | 1.43 | 0.005000 |
|  | c17782.graph_c0 | transmembrane protein 120 homolog | 4.04 | 1.25 | 0.005022 |
|  | c22918.graph_c0 | cytochrome b5-like heme steroid-binding domain protein | 50 | 1.25 | 0.005097 |
|  | c38780.graph_c0 | chlorophyll a-b binding protein chloroplastic | 21.11 | 1.64 | 0.005161 |
|  | c17992.graph_c0 | upf0047 protein | 8.95 | 1.29 | 0.005242 |
|  | c40671.graph_c0 | proline-rich family protein | 3.5 | 1.48 | 0.005284 |
|  | c31719.graph_c0 | cinnamyl alcohol dehydrogenase | 6.48 | 1.23 | 0.005310 |
|  | c37530.graph_c0 | caffeoyl- o-methyltransferase | 47.01 | 1.29 | 0.005377 |
|  | c28763.graph_c1 | neurofilament heavy polypeptide isoform x2 | 3.76 | 1.41 | 0.005435 |
|  | c15576.graph_c0 | probable aldo-keto reductase 2 | 36.28 | 1.28 | 0.005438 |
|  | c33233.graph_c1 | cytochrome p450 cyp94a1 family protein | 37.5 | 1.21 | 0.005446 |
|  | c24853.graph_c0 | wpp domain-interacting protein 1-like | 4.15 | 1.37 | 0.005472 |
|  | c38739.graph_c0 | glyceraldehyde-3-phosphate dehydrogenase chloroplastic | 39.8 | 1.43 | 0.005493 |
|  | c38771.graph_c0 | photosystem i reaction center subunit chloroplastic-like | 16.67 | 1.56 | 0.005566 |
|  | c40031.graph_c0 | aldehyde dehydrogenase family 2 member c4 | 62.02 | 1.38 | 0.005579 |
|  | c21669.graph_c0 | vacuolar-processing enzyme | 4.34 | 1.42 | 0.005604 |
|  | c15483.graph_c0 | eukaryotic translation initiation factor 6-2 | 31.4 | 1.22 | 0.005619 |
|  | c42598.graph_c0 | uncharacterized protein | 65.31 | 1.46 | 0.005644 |
|  | c33051.graph_c0 | d-amino-acid chloroplastic | 18.42 | 1.20 | 0.005678 |
|  | c36783.graph_c1 | map3k epsilon protein kinase 1-like isoform x2 | 8.64 | 1.35 | 0.005694 |
|  | c39003.graph_c0 | uncharacterized protein | 13.11 | 1.28 | 0.005702 |
|  | c38849.graph_c0 | fasciclin-like arabinogalactan protein 10 | 34.75 | 1.35 | 0.005833 |
|  | c41361.graph_c0 | caffeoyl- o-methyltransferase | 39.29 | 1.55 | 0.005928 |
|  | c24225.graph_c1 | hypothetical protein Ccrd_000223 | 47.37 | 1.41 | 0.005930 |
|  | c23454.graph_c0 | cysteine-rich repeat secretory protein 55-like | 16.48 | 1.27 | 0.005985 |
|  | c38886.graph_c0 | atp synthase delta chloroplastic | 13.1 | 1.40 | 0.005997 |
|  | c38344.graph_c0 | gdsl esterase lipase at5g45670-like | 27.99 | 1.52 | 0.006005 |
|  | c25447.graph_c0 | probable inactive receptor kinase at4g23740 | 1.46 | 1.36 | 0.006088 |
|  | c38825.graph_c0 | superoxide dismutase | 26.13 | 1.23 | 0.006099 |
|  | c17486.graph_c0 | peptidyl-prolyl cis-trans isomerase fkbp62-like | 40.32 | 1.25 | 0.006101 |
|  | c17915.graph_c0 | betaine aldehyde dehydrogenase | 44.25 | 1.23 | 0.006148 |
|  | c39401.graph_c0 | cytochrome p450 | 14.98 | 1.44 | 0.006255 |
|  | c24477.graph_c0 | gmp synthase | 14.08 | 1.43 | 0.006305 |
|  | c39575.graph_c0 | uncharacterized protein LOC105169991 | 42.65 | 2.37 | 0.006393 |
|  | c23763.graph_c0 | aldehyde dehydrogenase family 2 member mitochondrial | 38.1 | 1.22 | 0.006406 |
|  | c38563.graph_c1 | glutathione s-transferase l3-like | 40.77 | 1.22 | 0.006495 |
|  | c16011.graph_c0 | chlorophyll a-b binding protein chloroplastic | 22.8 | 1.45 | 0.006506 |
|  | c39098.graph_c0 | sedoheptulose- - chloroplastic | 21.63 | 1.38 | 0.006531 |
|  | c33246.graph_c0 | PREDICTED: uncharacterized protein LOC103930936 | 2.21 | 1.25 | 0.006539 |
|  | c38376.graph_c0 | chaperone protein 1 | 21.62 | 1.38 | 0.006595 |
|  | c38571.graph_c0 | aldo-keto reductase family 4 member c9-like | 36.71 | 1.20 | 0.006656 |
|  | c25186.graph_c0 | gdsl esterase lipase at3g48460 | 2.91 | 1.59 | 0.006737 |
|  | c40695.graph_c0 | vacuolar protein sorting-associated protein 32 homolog 2 | 34.86 | 1.30 | 0.006745 |
|  | c33797.graph_c0 | ubiquitin thioesterase otu1 | 21.53 | 1.34 | 0.006753 |
|  | c40052.graph_c0 | PREDICTED: uncharacterized protein LOC105160472 | 7.41 | 1.47 | 0.006825 |
|  | c29484.graph_c1 | bet v i domain-containing protein | 26.42 | 1.31 | 0.006889 |
|  | c39455.graph_c0 | apolipoprotein d-like | 50.27 | 1.92 | 0.006918 |
|  | c39108.graph_c0 | fasciclin-like arabinogalactan protein 9 | 12.45 | 1.34 | 0.006945 |
|  | c17343.graph_c0 | plant vap | 39.49 | 1.22 | 0.006985 |
|  | c39621.graph_c0 | bahd acyltransferase dcr | 16.42 | 1.30 | 0.006986 |
|  | c34320.graph_c3 | monocopper oxidase-like protein sku5 | 31.43 | 1.22 | 0.007016 |
|  | c38912.graph_c0 | cysteine proteinase 3 | 23.97 | 1.33 | 0.007058 |
|  | c44225.graph_c0 | translational elongation factor 1 subunit bbeta | 29.73 | 1.48 | 0.007095 |
|  | c42698.graph_c0 | protein opi10 homolog | 27.23 | 1.62 | 0.007242 |
|  | c48012.graph_c0 | atp-dependent dna helicase 2 subunit ku80 | 12.95 | 1.32 | 0.007282 |
|  | c18960.graph_c0 | replication factor c subunit 1 | 9.91 | 1.26 | 0.007329 |
|  | c30503.graph_c0 | uncharacterized protein | 8.33 | 1.35 | 0.007355 |
|  | c40756.graph_c0 | protein tic chloroplastic | 15.77 | 1.36 | 0.007365 |
|  | c17807.graph_c0 | glycoside catalytic domain-containing protein | 23.5 | 1.33 | 0.007433 |
|  | c32866.graph_c0 | glutathione s-transferase chloride c-terminal | 14.29 | 1.26 | 0.007438 |
|  | c40468.graph_c0 | mitochondrial import inner membrane translocase subunit tim22-2 | 5 | 1.32 | 0.007527 |
|  | c35246.graph_c0 | bifunctional purple acid phosphatase 26 | 11.69 | 1.29 | 0.007556 |
|  | c21473.graph_c0 | hypothetical protein Ccrd_022466 | 8.14 | 1.22 | 0.007659 |
|  | c16772.graph_c0 | alpha beta hydrolase domain-containing protein 11 | 8.33 | 1.35 | 0.007680 |
|  | c33398.graph_c0 | feruloyl ortho-hydroxylase 1 | 10.55 | 1.65 | 0.007805 |
|  | c37842.graph_c0 | probable lrr receptor-like serine threonine-protein kinase at1g67720 | 5.63 | 1.30 | 0.007860 |
|  | c24935.graph_c2 | rna polymerase ii degradation factor 1 | 11.5 | 1.32 | 0.007895 |
|  | c16363.graph_c1 | Protein of unknown function DUF1191 | 5.36 | 1.51 | 0.007901 |
|  | c29742.graph_c0 | alpha-galactosidase | 9.8 | 1.49 | 0.007923 |
|  | c40096.graph_c0 | serine carboxypeptidase 1 | 4.25 | 1.36 | 0.008014 |
|  | c39072.graph_c0 | 21 kda | 5.49 | 1.60 | 0.008044 |
|  | c40316.graph_c0 | serine racemase | 52.54 | 1.21 | 0.008248 |
|  | c24424.graph_c1 | adenylate kinase 4 | 55.25 | 1.22 | 0.008278 |
|  | c23128.graph_c0 | 50s ribosomal protein chloroplastic | 16.23 | 1.59 | 0.008292 |
|  | c39649.graph_c0 | histidine-containing phosphotransfer protein 1-like | 24.34 | 1.21 | 0.008325 |
|  | c16219.graph_c0 | tubulin-folding cofactor a | 46.9 | 1.41 | 0.008339 |
|  | c36170.graph_c1 | non-heme dioxygenase n-terminal domain-containing protein | 17.56 | 1.23 | 0.008468 |
|  | c44986.graph_c0 | harpin-induced family protein | 6.4 | 1.30 | 0.008548 |
|  | c24606.graph_c0 | desi-like protein at4g17486 isoform x2 | 9.91 | 1.24 | 0.008622 |
|  | c34718.graph_c1 | non-specific lipid-transfer protein | 35.04 | 1.67 | 0.008632 |
|  | c42372.graph_c0 | bet v i domain-containing protein | 23.53 | 1.34 | 0.008650 |
|  | c15288.graph_c0 | novel plant snare 11 | 7.6 | 1.22 | 0.008674 |
|  | c42340.graph_c0 | oxysterol-binding protein 1d | 2.45 | 1.41 | 0.008689 |
|  | c52377.graph_c0 | uncharacterized protein | 8.64 | 1.21 | 0.008893 |
|  | c17521.graph_c0 | macrophage migration inhibitory factor homolog | 9.52 | 1.56 | 0.008904 |
|  | c39256.graph_c0 | 30s ribosomal protein chloroplastic | 11.7 | 1.24 | 0.008964 |
|  | c15936.graph_c0 | probable calcium-binding protein cml36 | 5.86 | 1.44 | 0.008990 |
|  | c41113.graph_c0 | arabinogalactan protein | 23.38 | 1.32 | 0.008998 |
|  | c31253.graph_c1 | hypothetical protein Ccrd_008759 | 40 | 1.30 | 0.009000 |
|  | c33752.graph_c1 | transcription initiation factor tfiid subunit 4b-like | 4.95 | 1.68 | 0.009003 |
|  | c38939.graph_c0 | 50s ribosomal protein chloroplastic | 13.55 | 1.36 | 0.009056 |
|  | c39068.graph_c0 | 14-3-3-like family protein | 64.57 | 1.23 | 0.009081 |
|  | c38778.graph_c0 | atp synthase subunit b chloroplastic | 23.29 | 1.33 | 0.009155 |
|  | c39712.graph_c0 | 50s ribosomal protein chloroplastic | 12.25 | 1.37 | 0.009165 |
|  | c34491.graph_c0 | polyadenylate-binding protein rbp45 isoform x2 | 16.57 | 1.27 | 0.009236 |
|  | c40703.graph_c0 | phosphatidylglycerol phosphatidylinositol transfer protein | 18.63 | 1.48 | 0.009247 |
|  | c15802.graph_c0 | protein yipf1 homolog | 5.34 | 1.25 | 0.009339 |
|  | c34828.graph_c0 | probable aquaporin pip2-5 | 19.78 | 1.77 | 0.009444 |
|  | c41882.graph_c0 | glutelin type-b 5-like | 12.92 | 1.25 | 0.009474 |
|  | c23452.graph_c0 | phosphoglyceride transfer family protein isoform 1 | 44.63 | 1.46 | 0.009483 |
|  | c39943.graph_c0 | snf1-related protein kinase regulatory subunit gamma-1 | 16.99 | 1.27 | 0.009670 |
|  | c27438.graph_c0 | pi-plc x domain-containing protein at5g67130-like isoform x1 | 10.51 | 1.35 | 0.009683 |
|  | c42599.graph_c0 | chaperonin 60 subunit beta chloroplastic | 36.12 | 1.28 | 0.009742 |
|  | c41003.graph_c0 | expansin-like b1 | 32.03 | 1.37 | 0.009763 |
|  | c39529.graph_c0 | calcium sensing chloroplastic | 23.72 | 1.36 | 0.009817 |
|  | c22412.graph_c0 | chorismate mutase chloroplastic | 11.36 | 1.24 | 0.009858 |
|  | c39792.graph_c0 | isopentenyl-diphosphate delta-isomerasei | 45.8 | 1.39 | 0.009929 |
|  | c37344.graph_c0 | prp27-like protein | 21.17 | 1.33 | 0.009946 |
|  | c42166.graph_c0 | remorin-like | 29.63 | 1.22 | 0.010009 |
|  | c26957.graph_c0 | uncharacterized protein | 24.01 | 1.24 | 0.010033 |
|  | c19048.graph_c0 | glutathione s-transferase l3 | 23.23 | 1.26 | 0.010036 |
|  | c41197.graph_c0 | protein of unknown function DUF4408 | 5.25 | 1.65 | 0.010078 |
|  | c29137.graph_c0 | heat shock transcription factor a1c1 | 1.91 | 1.27 | 0.010095 |
|  | c31085.graph_c0 | vesicle transport v-snare 13-like | 9.95 | 1.45 | 0.010222 |
|  | c38971.graph_c0 | vacuolar processing enzyme | 7.66 | 0.40 | 0.000003 |
|  | c42603.graph_c0 | mitochondrial amidoxime reducing component 2-like | 5.54 | 0.76 | 0.000010 |
|  | c17360.graph_c0 | thaumatin-like protein 1a | 7.98 | 0.46 | 0.000018 |
|  | c37727.graph_c0 | cinnamoyl- reductase family protein | 11.28 | 0.73 | 0.000028 |
|  | c39315.graph_c0 | protein aspartic protease in guard cell 2-like | 23.23 | 0.63 | 0.000044 |
|  | c41861.graph_c0 | mlp-like protein 423 | 52.63 | 0.71 | 0.000046 |
|  | c26369.graph_c0 | beta- -glucanase | 6.42 | 0.45 | 0.000046 |
|  | c42781.graph_c0 | pyrroline-5-carboxylate synthetase isoform 1 | 20.5 | 0.59 | 0.000048 |
|  | c40554.graph_c0 | wd repeat-containing protein lwd1 | 3.6 | 0.55 | 0.000049 |
|  | c35082.graph_c0 | mediator of rna polymerase ii transcription subunit 15 isoform x2 | 5.32 | 0.70 | 0.000066 |
|  | c24474.graph_c0 | glucose-6-phosphate 1- chloroplastic | 18.75 | 0.76 | 0.000073 |
|  | c38733.graph_c0 | nucleotide- alpha-beta plait | 75 | 0.54 | 0.000073 |
|  | c39807.graph_c0 | sts14 protein | 35.93 | 0.66 | 0.000098 |
|  | c24972.graph_c0 | udp-d-apiose udp-d-xylose synthase 2 | 61.74 | 0.82 | 0.000105 |
|  | c41012.graph_c0 | protein cdi-like | 7.66 | 0.56 | 0.000109 |
|  | c38946.graph_c0 | 40s ribosomal protein s26-3-like | 50.77 | 0.73 | 0.000111 |
|  | c38112.graph_c0 | k(+) efflux antiporter chloroplastic-like | 2.22 | 0.69 | 0.000120 |
|  | c37186.graph_c0 | hypothetical chloroplast rf21 | 4.2 | 0.83 | 0.000122 |
|  | c38757.graph_c0 | protein aspartic protease in guard cell 2-like | 40.98 | 0.82 | 0.000135 |
|  | c23142.graph_c0 | endoglucanase 8 | 6.68 | 0.63 | 0.000139 |
|  | c41744.graph_c0 | 50s ribosomal protein l19- chloroplastic-like | 7.02 | 0.64 | 0.000167 |
|  | c23508.graph_c0 | pyruvate cytosolic isozyme | 49.8 | 0.78 | 0.000170 |
|  | c16103.graph_c0 | -beta-fructan 1f-fructosyltransferase | 28.83 | 0.54 | 0.000194 |
|  | c17479.graph_c1 | bet v i domain-containing protein | 54.39 | 0.78 | 0.000196 |
|  | c38508.graph_c1 | pyruvate decarboxylase 1 | 44.14 | 0.68 | 0.000197 |
|  | c30146.graph_c0 | subtilisin-like protease | 6.03 | 0.78 | 0.000232 |
|  | c29620.graph_c0 | protein exordium-like 3 | 10.66 | 0.71 | 0.000247 |
|  | c39080.graph_c0 | subtilisin-like protease | 18.86 | 0.79 | 0.000277 |
|  | c16587.graph_c0 | probable carbohydrate esterase at4g34215 | 8.83 | 0.59 | 0.000290 |
|  | c30904.graph_c0 | nadp-malic enzyme | 23.8 | 0.83 | 0.000291 |
|  | c41564.graph_c0 | gdp-mannose transporter gonst4 | 4.35 | 0.68 | 0.000304 |
|  | c39275.graph_c0 | cystathionine gamma synthase | 23.52 | 0.78 | 0.000315 |
|  | c15823.graph_c1 | actin | 67.29 | 0.75 | 0.000333 |
|  | c39043.graph_c0 | barwin-like endoglucanase | 8.11 | 0.79 | 0.000338 |
|  | c16077.graph_c0 | barwin-like endoglucanase | 9.89 | 0.50 | 0.000340 |
|  | c32125.graph_c0 | monodehydroascorbate reductase | 47.34 | 0.83 | 0.000343 |
|  | c35484.graph_c0 | gdsl esterase lipase exl3-like | 25.17 | 0.59 | 0.000355 |
|  | c35385.graph_c0 | iaa-amino acid hydrolase ilr1-like 2 | 20.77 | 0.75 | 0.000390 |
|  | c39036.graph_c0 | protein translation factor sui1 homolog 2-like | 21.05 | 0.54 | 0.000411 |
|  | c39879.graph_c0 | pathogenesis-related protein 1 | 15.43 | 0.63 | 0.000437 |
|  | c39639.graph_c0 | phospho-2-dehydro-3-deoxyheptonate aldolase chloroplastic | 33.33 | 0.79 | 0.000480 |
|  | c31140.graph_c0 | angio-associated migratory cell protein | 16.62 | 0.82 | 0.000494 |
|  | c40705.graph_c0 | aspartic proteinase nepenthesin-2-like | 12.34 | 0.81 | 0.000495 |
|  | c35269.graph_c0 | probable pyridoxal biosynthesis protein | 18.75 | 0.53 | 0.000500 |
|  | c38991.graph_c0 | protein p21-like | 41.7 | 0.56 | 0.000520 |
|  | c16546.graph_c0 | lysosomal beta glucosidase-like | 18.07 | 0.77 | 0.000557 |
|  | c34690.graph_c1 | monosaccharide-sensing protein 2 | 11.03 | 0.69 | 0.000557 |
|  | c40489.graph_c0 | bet v i domain-containing protein | 54.43 | 0.81 | 0.000565 |
|  | c38370.graph_c0 | duf21 domain-containing protein at4g14240-like | 14.13 | 0.81 | 0.000581 |
|  | c31342.graph_c0 | probable xyloglucan endotransglucosylase hydrolase protein b | 43.72 | 0.58 | 0.000588 |
|  | c32250.graph_c1 | gdsl-motif lipase hydrolase family protein | 2.32 | 0.68 | 0.000600 |
|  | c38461.graph_c1 | dolichyl-diphosphooligosaccharide--protein glycosyltransferase subunit stt3a | 3.39 | 0.68 | 0.000604 |
|  | c17798.graph_c0 | fasciclin-like arabinogalactan protein 2 | 17.65 | 0.70 | 0.000609 |
|  | c39521.graph_c0 | leucine-rich repeat receptor-like protein kinase pxc1 | 10.8 | 0.75 | 0.000623 |
|  | c36087.graph_c0 | 3 -n-debenzoyl-2 -deoxytaxol n-benzoyltransferase | 5.87 | 0.38 | 0.000628 |
|  | c23220.graph_c0 | glyceraldehyde-3-phosphate cytosolic-like | 62.99 | 0.65 | 0.000651 |
|  | c34925.graph_c0 | inosine-uridine preferring nucleoside hydrolase family protein isoform 1 | 8.92 | 0.77 | 0.000673 |
|  | c41686.graph_c0 | probable thylakoid lumen protein sll1769 isoform x1 | 27.86 | 0.79 | 0.000687 |
|  | c32207.graph_c0 | homeobox-leucine zipper protein glabra 2 | 1.61 | 0.78 | 0.000695 |
|  | c40319.graph_c0 | phosphoenolpyruvate carboxykinase | 30.97 | 0.83 | 0.000699 |
|  | c38819.graph_c0 | glycine-rich protein 2-like | 34.62 | 0.58 | 0.000729 |
|  | c40822.graph_c0 | 11-s seed storage plant | 51.67 | 0.65 | 0.000766 |
|  | c40055.graph_c0 | beta expansin precursor | 12.73 | 0.47 | 0.000807 |
|  | c39832.graph_c0 | nadh dehydrogenase | 36.31 | 0.65 | 0.000842 |
|  | c39153.graph_c0 | protein spiral1-like 1 | 41.59 | 0.50 | 0.000860 |
|  | c41840.graph_c0 | cyclin-d1-binding protein 1 homolog | 4.93 | 0.81 | 0.000869 |
|  | c34919.graph_c1 | zinc finger ccch domain-containing protein 53-like isoform x1 | 6.04 | 0.67 | 0.000870 |
|  | c38513.graph_c0 | probable lrr receptor-like serine threonine-protein kinase at1g53430 | 4.81 | 0.71 | 0.000916 |
|  | c39716.graph_c0 | glycine-rich rna-binding protein mitochondrial-like | 40.56 | 0.80 | 0.000926 |
|  | c17737.graph_c0 | lipid-transfer protein dir1 | 20 | 0.55 | 0.000980 |
|  | c57075.graph_c0 | uncharacterized protein | 36 | 0.62 | 0.000982 |
|  | c38520.graph_c0 | cyclopropane-fatty-acyl-phospholipid synthase | 6.21 | 0.72 | 0.000995 |
|  | c27781.graph_c0 | glucan endo- -beta-glucosidase 8 | 20.04 | 0.76 | 0.001006 |
|  | c16057.graph_c0 | probable polygalacturonase isoform x2 | 24.4 | 0.68 | 0.001006 |
|  | c16000.graph_c0 | bifunctional inhibitor plant lipid transfer protein seed storage helical domain-containing protein | 29.17 | 0.68 | 0.001011 |
|  | c32185.graph_c0 | actin-7-like | 18.26 | 0.78 | 0.001011 |
|  | c38086.graph_c0 | udp-glucose:flavonoid 3-o-glucosyltransferase | 20.08 | 0.51 | 0.001033 |
|  | c29040.graph_c0 | 50s ribosomal protein l30 | 30.63 | 0.83 | 0.001051 |
|  | c39448.graph_c0 | uncharacterized protein | 13.29 | 0.55 | 0.001056 |
|  | c15986.graph_c1 | protein aspartic protease in guard cell 2-like | 30.75 | 0.80 | 0.001087 |
|  | c39617.graph_c0 | pectinesterase family protein | 15.58 | 0.55 | 0.001114 |
|  | c33522.graph_c0 | nad -binding domain-containing partial | 21.43 | 0.76 | 0.001121 |
|  | c39838.graph_c0 | Protein of unknown function DUF3411 | 7.95 | 0.76 | 0.001141 |
|  | c40106.graph_c0 | Unknown | 14.66 | 0.47 | 0.001181 |
|  | c42831.graph_c0 | protein too many mouths | 14.88 | 0.73 | 0.001217 |
|  | c38744.graph_c0 | sucrose:sucrose 1-fructosyl transferase | 28.01 | 0.65 | 0.001228 |
|  | c38954.graph_c0 | 50s ribosomal protein chloroplastic-like | 10.19 | 0.68 | 0.001251 |
|  | c15950.graph_c0 | protein yls9-like | 33.02 | 0.83 | 0.001280 |
|  | c39843.graph_c0 | galactose-binding domain-like partial | 23.37 | 0.79 | 0.001285 |
|  | c26249.graph_c0 | k+-h+ exchange-like protein | 2.66 | 0.80 | 0.001297 |
|  | c35983.graph_c0 | transcription factor bhlh130-like | 5.41 | 0.60 | 0.001303 |
|  | c24689.graph_c0 | zinc finger ccch domain-containing protein 53-like isoform x1 | 6.19 | 0.78 | 0.001374 |
|  | c38572.graph_c1 | pleiotropic drug resistance protein 1 | 19.48 | 0.75 | 0.001387 |
|  | c41002.graph_c0 | protein light-dependent short hypocotyls 10-like | 31.14 | 0.77 | 0.001400 |
|  | c39995.graph_c0 | lysosomal beta glucosidase-like | 30.95 | 0.74 | 0.001419 |
|  | c25432.graph_c0 | hypothetical protein Ccrd_012362 | 8.93 | 0.83 | 0.001478 |
|  | c40950.graph_c0 | 50s ribosomal protein l3- chloroplastic | 24.84 | 0.83 | 0.001517 |
|  | c40320.graph_c0 | dystrophia myotonica wd repeat-containing | 5.8 | 0.82 | 0.001518 |
|  | c15982.graph_c0 | nucleobase-ascorbate transporter 6 | 10.78 | 0.80 | 0.001555 |
|  | c29773.graph_c0 | autophagy-related protein 101 | 9.49 | 0.78 | 0.001576 |
|  | c13307.graph_c0 | histone h2b | 17.78 | 0.49 | 0.001593 |
|  | c18076.graph_c0 | 1-aminocyclopropane-1-carboxylate oxidase 1 | 48.84 | 0.67 | 0.001606 |
|  | c34712.graph_c0 | adenosylhomocysteinase 1 | 63.92 | 0.82 | 0.001616 |
|  | c35628.graph_c0 | dirigent protein 22-like | 21.12 | 0.72 | 0.001625 |
|  | c40090.graph_c0 | bet v i domain-containing protein | 13.33 | 0.69 | 0.001637 |
|  | c35560.graph_c0 | ctp synthase-like | 9.98 | 0.72 | 0.001678 |
|  | c46966.graph_c0 | bet v i domain-containing protein | 37.68 | 0.83 | 0.001710 |
|  | c39336.graph_c0 | alpha- -glucan-protein synthase | 70 | 0.77 | 0.001727 |
|  | c40876.graph_c0 | protein rcc2 homolog | 18.48 | 0.80 | 0.001729 |
|  | c24409.graph_c1 | tho complex subunit 4a | 8.26 | 0.81 | 0.001735 |
|  | c38062.graph_c0 | transducin family protein wd-40 repeat family isoform 2 | 5.08 | 0.82 | 0.001753 |
|  | c38972.graph_c0 | protein aspartic protease in guard cell 1-like | 19.25 | 0.80 | 0.001771 |
|  | c16900.graph_c0 | pyruvate kinase isozyme chloroplastic isoform x1 | 14.06 | 0.77 | 0.001805 |
|  | c19035.graph_c0 | act domain-containing protein | 13.77 | 0.78 | 0.001846 |
|  | c30666.graph_c0 | protein aspartic protease in guard cell 2-like | 34.3 | 0.76 | 0.001858 |
|  | c42609.graph_c0 | dctp pyrophosphatase 1-like | 14.81 | 0.63 | 0.001875 |
|  | c32416.graph_c0 | eukaryotic translation initiation factor 3 subunit b | 21.38 | 0.81 | 0.001876 |
|  | c25413.graph_c0 | pi-plc x domain-containing protein at5g67130-like | 3.47 | 0.69 | 0.001877 |
|  | c35553.graph_c0 | bifunctional riboflavin kinase fmn phosphatase-like | 7.91 | 0.63 | 0.001879 |
|  | c39074.graph_c0 | uncharacterized protein | 47.37 | 0.66 | 0.001939 |
|  | c39070.graph_c0 | subtilisin-like protease | 20.6 | 0.79 | 0.002061 |
|  | c16604.graph_c0 | hypothetical protein Ccrd_023437, partial | 20.47 | 0.73 | 0.002125 |
|  | c38404.graph_c0 | PREDICTED: uncharacterized protein LOC8262870 isoform X2 | 2.36 | 0.59 | 0.002230 |
|  | c24245.graph_c1 | type i cytoskeletal 10 | 6.94 | 0.78 | 0.002240 |
|  | c29631.graph_c0 | transmembrane 9 superfamily member 11 | 11.57 | 0.75 | 0.002266 |
|  | c38505.graph_c0 | isoform 1 | 12.63 | 0.75 | 0.002274 |
|  | c24185.graph_c0 | glycerophosphodiester phosphodiesterase gdpd6 | 23.66 | 0.82 | 0.002277 |
|  | c39726.graph_c0 | glucan endo- -beta-glucosidase-like | 12.03 | 0.76 | 0.002307 |
|  | c17831.graph_c0 | rab-gtpase-tbc domain-containing protein | 8.23 | 0.70 | 0.002344 |
|  | c43053.graph_c0 | probable cytosolic iron-sulfur protein assembly protein ciao1 homolog | 20.33 | 0.65 | 0.002364 |
|  | c16416.graph_c0 | phosphatase chloroplastic | 23.96 | 0.73 | 0.002375 |
|  | c40765.graph_c0 | nadph:quinone oxidoreductase | 11.73 | 0.63 | 0.002459 |
|  | c41289.graph_c0 | heparanase-like protein 1 | 12.08 | 0.77 | 0.002521 |
|  | c36623.graph_c0 | dynamin-related protein 3a isoform 1 | 10.88 | 0.68 | 0.002543 |
|  | c36455.graph_c0 | probable leucine-rich repeat receptor-like protein kinase at1g35710 | 15.7 | 0.76 | 0.002601 |
|  | c31558.graph_c0 | u4 tri-snrnp-associated protein 2-like | 4.95 | 0.78 | 0.002620 |
|  | c41317.graph_c0 | probable fructose-bisphosphate aldolase chloroplastic | 38.29 | 0.83 | 0.002659 |
|  | c24253.graph_c0 | beta-galactosidase 8 isoform 1 | 26.88 | 0.78 | 0.002751 |
|  | c36405.graph_c0 | associated molecule with the sh3 domain of stam 3 isoform 1 | 4.51 | 0.80 | 0.002751 |
|  | c16387.graph_c0 | d-3-phosphoglycerate dehydrogenase chloroplastic-like | 37.46 | 0.79 | 0.002778 |
|  | c41842.graph_c0 | protein light-dependent short hypocotyls 1-like | 19.38 | 0.63 | 0.002873 |
|  | c40239.graph_c0 | bifunctional l-3-cyanoalanine synthase cysteine synthase mitochondrial | 36.02 | 0.75 | 0.002878 |
|  | c18559.graph_c0 | mitochondrial import receptor subunit tom20-like | 18.54 | 0.72 | 0.002886 |
|  | c25410.graph_c0 | protein quirky-like | 4.4 | 0.80 | 0.002919 |
|  | c39637.graph_c0 | small nuclear ribonucleoprotein sm d3 | 20.16 | 0.76 | 0.003030 |
|  | c33746.graph_c0 | atp binding microtubule motor family protein isoform 1 | 8.76 | 0.53 | 0.003056 |
|  | c35612.graph_c0 | regulatory protein npr1-like | 13.22 | 0.71 | 0.003056 |
|  | c39129.graph_c0 | PREDICTED: LOW QUALITY PROTEIN: uncharacterized protein LOC107004534 | 57.18 | 0.73 | 0.003116 |
|  | c42048.graph_c0 | erlin-2-b | 23.98 | 0.82 | 0.003153 |
|  | c41595.graph_c0 | embryonic protein dc-8 | 40.54 | 0.81 | 0.003175 |
|  | c41927.graph_c0 | uncharacterized gtp-binding protein at5g64813-like | 3.32 | 0.78 | 0.003192 |
|  | c24975.graph_c0 | glycine dehydrogenase mitochondrial | 28.48 | 0.73 | 0.003203 |
|  | c16238.graph_c0 | probable tyrosine-protein phosphatase at1g05000 | 14.67 | 0.63 | 0.003232 |
|  | c30088.graph_c0 | mitotic checkpoint protein | 4.4 | 0.83 | 0.003248 |
|  | c36055.graph_c0 | aaa- cdc48 protein | 75.57 | 0.70 | 0.003267 |
|  | c22846.graph_c0 | cysteine--trna cytoplasmic isoform x2 | 9.49 | 0.81 | 0.003324 |
|  | c42466.graph_c0 | rrna methyltransferase mitochondrial | 5.81 | 0.67 | 0.003354 |
|  | c15566.graph_c0 | purple acid phosphatase 2 | 2.2 | 0.72 | 0.003370 |
|  | c37653.graph_c1 | glycoside catalytic domain-containing protein | 17.56 | 0.65 | 0.003479 |
|  | c39823.graph_c0 | inositol-phosphate phosphatase | 41.85 | 0.78 | 0.003523 |
|  | c15677.graph_c0 | nadh dehydrogenase | 3.96 | 0.83 | 0.003539 |
|  | c23226.graph_c2 | ribulose bisphosphate carboxylase oxygenase chloroplastic-like | 23.68 | 0.58 | 0.003593 |
|  | c38044.graph_c0 | alcohol c-terminal | 73.86 | 0.79 | 0.003611 |
|  | c35813.graph_c0 | 40s ribosomal protein s25 | 45.37 | 0.72 | 0.003617 |
|  | c17274.graph_c0 | nuclear transcription factor y subunit c-9-like isoform x1 | 18.22 | 0.83 | 0.003690 |
|  | c25524.graph_c0 | fatty acid amide hydrolase | 13.44 | 0.79 | 0.003730 |
|  | c40477.graph_c0 | agmatine deiminase | 6.4 | 0.74 | 0.003734 |
|  | c37464.graph_c0 | probable lrr receptor-like serine threonine-protein kinase rfk1 isoform x1 | 2.08 | 0.45 | 0.003790 |
|  | c15976.graph_c0 | osmotin-like protein | 34.3 | 0.81 | 0.003904 |
|  | c39127.graph_c0 | glucomannan 4-beta-mannosyltransferase 2-like | 11.92 | 0.79 | 0.003977 |
|  | c38875.graph_c0 | aspartic proteinase-like | 25.15 | 0.81 | 0.003992 |
|  | c34493.graph_c0 | ap-2 complex subunit alpha-1-like isoform x3 | 14.85 | 0.68 | 0.004004 |
|  | c39156.graph_c0 | sulfate transporter -like | 5.42 | 0.74 | 0.004020 |
|  | c36105.graph_c0 | had-like domain-containing protein | 4.03 | 0.53 | 0.004064 |
|  | c43314.graph_c0 | non-heme dioxygenase n-terminal domain-containing protein | 7.32 | 0.73 | 0.004195 |
|  | c35777.graph_c0 | dicer-like protein 4 isoform x2 | 4.95 | 0.79 | 0.004228 |
|  | c26884.graph_c1 | zinc finger ccch domain-containing protein 34-like | 2.04 | 0.79 | 0.004232 |
|  | c38859.graph_c0 | 60s ribosomal protein l13a-4 | 60.19 | 0.81 | 0.004287 |
|  | c42314.graph_c0 | actin-related protein 2 3 complex subunit 2a | 5.9 | 0.71 | 0.004304 |
|  | c43681.graph_c0 | isocitrate dehydrogenase | 11.58 | 0.76 | 0.004323 |
|  | c39677.graph_c0 | uncharacterized oxidoreductase at4g09670 | 34.35 | 0.75 | 0.004592 |
|  | c36997.graph_c0 | 50s ribosomal protein mitochondrial isoform x3 | 22.22 | 0.79 | 0.004659 |
|  | c42184.graph_c0 | endoglucanase 3-like | 18.84 | 0.83 | 0.004667 |
|  | c15995.graph_c0 | phosphomethylpyrimidine chloroplastic | 27.8 | 0.78 | 0.004733 |
|  | c34641.graph_c0 | peroxidase 55-like | 4.14 | 0.64 | 0.004782 |
|  | c27026.graph_c0 | acid phosphatase 1-like | 5.86 | 0.76 | 0.004799 |
|  | c42224.graph_c0 | chitin- type 1 | 20.51 | 0.79 | 0.004866 |
|  | c39775.graph_c0 | acid beta-fructofuranosidase-like | 4.02 | 0.72 | 0.004879 |
|  | c42230.graph_c0 | 39s ribosomal protein mitochondrial | 3.83 | 0.81 | 0.004907 |
|  | c17532.graph_c0 | pathogenesis-related family protein | 13.66 | 0.81 | 0.004913 |
|  | c39062.graph_c0 | glycine-rich protein 2-like | 34.88 | 0.75 | 0.005007 |
|  | c32756.graph_c0 | probable methyltransferase pmt5 | 5.38 | 0.75 | 0.005026 |
|  | c53185.graph_c0 | tetrahydrocannabinolic acid synthase-like | 19.13 | 0.77 | 0.005034 |
|  | c38976.graph_c0 | zinc finger ccch domain-containing protein 20-like | 6.67 | 0.78 | 0.005079 |
|  | c34476.graph_c0 | glutamate decarboxylase | 19.76 | 0.82 | 0.005120 |
|  | c42462.graph_c0 | lignin-forming anionic peroxidase-like | 19.75 | 0.64 | 0.005211 |
|  | c49975.graph_c0 | phospholipase d delta | 8.63 | 0.60 | 0.005212 |
|  | c39550.graph_c0 | 40s ribosomal protein s29 | 75 | 0.79 | 0.005220 |
|  | c33778.graph_c0 | ultraviolet-b receptor uvr8 | 29.35 | 0.75 | 0.005300 |
|  | c20985.graph_c0 | glycogen phosphorylase 1-like | 5.31 | 0.76 | 0.005304 |
|  | c31549.graph_c0 | global transcription factor group isoform 1 | 2.94 | 0.70 | 0.005330 |
|  | c42067.graph_c0 | regulatory npr5 -like protein | 18.06 | 0.82 | 0.005333 |
|  | c40913.graph_c0 | glucan endo- -beta-glucosidase 12 | 5.01 | 0.80 | 0.005459 |
|  | c16289.graph_c0 | polygalacturonase at1g48100-like | 9.57 | 0.74 | 0.005469 |
|  | c31876.graph_c1 | golgin subfamily a member 6-like protein 6 | 18.1 | 0.79 | 0.005477 |
|  | c33270.graph_c0 | mannosyl-oligosaccharide -alpha-mannosidase mns1-like isoform x1 | 8.97 | 0.79 | 0.005511 |
|  | c39228.graph_c0 | subtilisin-like protease | 11.1 | 0.73 | 0.005660 |
|  | c17771.graph_c0 | pentatricopeptide repeat-containing protein mitochondrial-like | 8.52 | 0.76 | 0.005844 |
|  | c41530.graph_c0 | clathrin interactor epsin 2 | 6.3 | 0.74 | 0.006102 |
|  | c37576.graph_c1 | p-loop containing nucleoside triphosphate hydrolases superfamily protein isoform 1 | 9.3 | 0.72 | 0.006123 |
|  | c38580.graph_c0 | g-type lectin s-receptor-like serine threonine-protein kinase at4g03230 | 5.49 | 0.63 | 0.006145 |
|  | c34493.graph_c2 | ap-2 complex subunit alpha-1-like | 20 | 0.75 | 0.006168 |
|  | c29293.graph_c0 | protein light dependent short hypocotyls 5 like | 14.16 | 0.39 | 0.006255 |
|  | c16361.graph_c0 | protein early responsive to dehydration 15 isoform x1 | 16.47 | 0.72 | 0.006295 |
|  | c16870.graph_c0 | csf-2 family protein | 63.16 | 0.74 | 0.006299 |
|  | c29288.graph_c2 | probable inactive receptor kinase at1g48480 | 20.3 | 0.74 | 0.006392 |
|  | c16488.graph_c0 | pectinesterase family protein | 19.85 | 0.83 | 0.006432 |
|  | c24825.graph_c0 | oligopeptide transporter 3 | 3.1 | 0.72 | 0.006438 |
|  | c31433.graph_c0 | feruloyl ortho-hydroxylase 1 | 36.16 | 0.75 | 0.006442 |
|  | c16166.graph_c0 | atp-dependent clp protease family protein | 7.31 | 0.76 | 0.006490 |
|  | c39182.graph_c0 | hmg1 2-like protein | 15.29 | 0.75 | 0.006491 |
|  | c41622.graph_c0 | membrane steroid-binding protein 2-like | 11.17 | 0.74 | 0.006512 |
|  | c31891.graph_c0 | berberine berberine-like protein | 3.46 | 0.63 | 0.006530 |
|  | c25049.graph_c0 | cellulose synthase a catalytic subunit 3 | 9.42 | 0.83 | 0.006729 |
|  | c23904.graph_c0 | d-aminoacid aminotransferase-like plp-dependent enzymes superfamily protein | 7.96 | 0.83 | 0.006738 |
|  | c38207.graph_c0 | upf0481 protein at3g47200-like | 3.54 | 0.82 | 0.006858 |
|  | c30876.graph_c0 | ribosomal protein l2 | 22.57 | 0.77 | 0.007002 |
|  | c16195.graph_c0 | stress response protein nst1-like | 6.7 | 0.82 | 0.007012 |
|  | c38827.graph_c0 | 14 kda proline-rich | 10.2 | 0.80 | 0.007025 |
|  | c37728.graph_c0 | probable quinone oxidoreductase | 60 | 0.79 | 0.007033 |
|  | c44005.graph_c0 | fact complex subunit ssrp1 | 10.24 | 0.77 | 0.007133 |
|  | c42683.graph_c0 | rrna-processing protein efg1 | 5.97 | 0.76 | 0.007283 |
|  | c15525.graph_c0 | serine acetyltransferase chloroplastic-like | 9.02 | 0.82 | 0.007289 |
|  | c39559.graph_c0 | hypersensitive-induced response protein 1 | 33.68 | 0.81 | 0.007304 |
|  | c25656.graph_c3 | glyceraldehyde-3-phosphate cytosolic | 46.47 | 0.78 | 0.007318 |
|  | c38851.graph_c0 | gamma-glutamylcyclotransferase at3g02910 | 13.11 | 0.78 | 0.007564 |
|  | c43472.graph_c0 | membrane protein of er body-like protein isoform x1 | 2.92 | 0.58 | 0.007624 |
|  | c28351.graph_c0 | pyruvate decarboxylase 1 | 22.55 | 0.73 | 0.007782 |
|  | c41831.graph_c0 | pyruvate kinase isozyme chloroplastic-like isoform x3 | 1.94 | 0.73 | 0.007787 |
|  | c23690.graph_c0 | protein transport protein sec61 subunit beta-like | 12.94 | 0.82 | 0.007794 |
|  | c38474.graph_c0 | ribosomal protein l22p l17e family protein | 13.5 | 0.83 | 0.007846 |
|  | c17621.graph_c0 | 50s ribosomal protein mitochondrial-like | 6.16 | 0.79 | 0.007853 |
|  | c21136.graph_c0 | probable adp-ribosylation factor gtpase-activating protein agd14 isoform x2 | 6.87 | 0.77 | 0.007925 |
|  | c40140.graph_c0 | cdp-diacylglycerol--glycerol-3-phosphate 3-phosphatidyltransferase 2-like | 5.92 | 0.82 | 0.008007 |
|  | c40646.graph_c0 | protein quirky-like | 12.45 | 0.80 | 0.008025 |
|  | c24951.graph_c0 | desi-like protein at4g17486 | 7.47 | 0.82 | 0.008073 |
|  | c37831.graph_c1 | abc transporter b family member 20 | 4.55 | 0.74 | 0.008112 |
|  | c26490.graph_c0 | hexokinase 3 | 9.78 | 0.74 | 0.008278 |
|  | c23094.graph_c0 | monosaccharide-sensing protein 2 | 7.37 | 0.60 | 0.008288 |
|  | c34690.graph_c0 | monosaccharide-sensing protein 2-like | 5.91 | 0.53 | 0.008361 |
|  | c17641.graph_c0 | pre-mrna-processing factor 39 | 9.14 | 0.74 | 0.008391 |
|  | c16224.graph_c0 | histone deacetylase 6 | 6.41 | 0.62 | 0.008411 |
|  | c17315.graph_c0 | fk506-binding protein 4 | 15.87 | 0.81 | 0.008442 |
|  | c40183.graph_c0 | cysteine proteinase inhibitor | 40.85 | 0.76 | 0.008479 |
|  | c40215.graph_c0 | extensin-2- partial | 29.29 | 0.68 | 0.008538 |
|  | c43549.graph_c0 | peptidyl-prolyl cis-trans isomerase family protein | 29.07 | 0.70 | 0.008636 |
|  | c41130.graph_c0 | g3bp-like protein | 12.22 | 0.82 | 0.008649 |
|  | c39201.graph_c0 | dead-box atp-dependent rna helicase 46 | 9.15 | 0.75 | 0.008718 |
|  | c36836.graph_c0 | Protein of unknown function DUF3475 | 5.9 | 0.76 | 0.008739 |
|  | c22849.graph_c0 | gpi ethanolamine phosphate transferase 1 | 1.02 | 0.80 | 0.008769 |
|  | c34820.graph_c0 | serine threonine-protein kinase | 13 | 0.66 | 0.008793 |
|  | c40331.graph_c0 | hypothetical protein Ccrd_018973 | 25.29 | 0.79 | 0.008873 |
|  | c15294.graph_c0 | uncharacterized membrane protein at1g75140-like | 6.06 | 0.82 | 0.009036 |
|  | c37269.graph_c1 | probable aquaporin pip-type 7a | 36.52 | 0.83 | 0.009048 |
|  | c27720.graph_c0 | nuclear pore complex | 4.96 | 0.77 | 0.009143 |
|  | c14854.graph_c0 | golgin candidate 5 | 6.72 | 0.72 | 0.009476 |
|  | c29586.graph_c0 | lysosomal pro-x carboxypeptidase-like | 5.14 | 0.81 | 0.009536 |
|  | c16164.graph_c1 | xyloglucan endotransglucosylase hydrolase | 28.21 | 0.69 | 0.009541 |
|  | c25159.graph_c0 | glucan endo- -beta-glucosidase 6 | 15.91 | 0.80 | 0.009554 |
|  | c40445.graph_c0 | nucleotide- alpha-beta plait | 29.44 | 0.83 | 0.009613 |
|  | c34550.graph_c2 | atp-dependent zinc metalloprotease ftsh mitochondrial-like | 11.44 | 0.81 | 0.010046 |
|  | c29630.graph_c0 | wd repeat-containing protein 26-like | 5.37 | 0.75 | 0.010304 |
|  | c15773.graph_c0 | probable small nuclear ribonucleoprotein f | 15.91 | 0.83 | 0.010335 |
|  | c34652.graph_c0 | diphthamide biosynthesis protein 2 | 4.12 | 0.82 | 0.010357 |
