# Supplementary material for: Quantitative Proteomics Analysis of Lettuce (Lactuca sativa L.) Reveals Molecular Basis-Associated Auxin and Photosynthesis with Bolting Induced by High Temperature
Source: Int J Mol Sci. 2018 Sep 28;19(10):2967. doi: 10.3390/ijms19102967 (PMC6213495; doi:10.3390/ijms19102967)
Supplement: Supplementary file 1 [file ijms-19-02967-s001.zip › ijms-336629 Supplementary/supplemental table S3.docx]

Supplemental Table S3 The information of proteins on the basis of one unique peptide with one spectra

| **No.** | **Accession no.** | **Protein name** | **Peptide** | **Name of spectra** | **No. of unique Peptides** | **No. of spectra** | **Spectra** |
| --- | --- | --- | --- | --- | --- | --- | --- |
| 1 | c41990.graph_c0 | rna-binding protein lupus la | dSTVIEVQGENIR | DSTVIEVQGENIR_-64_2_134453 | 1 | 1 |  |
| 2 | c41444.graph_c0 | gibberellin-regulated protein 1-like | acGTccAR | ACGTCCAR_-64_2_249405 | 1 | 1 |  |
| 3 | c38731.graph_c0 | 2fe-2s ferredoxin superfamily protein | aGScSScAGk | AGSCSSCAGK_-64_2_285994 | 1 | 1 |  |
| 4 | c38761.graph_c0 | photosystem i reaction center subunit chloroplastic | eGEPSIAPSLTLTGR | EGEPSIAPSLTLTGR_-64_2_24571 | 1 | 1 |  |
| 5 | c38822.graph_c0 | chlorophyll a-b binding protein chloroplastic | yPGGAFDPLGYSk | YPGGAFDPLGYSK_-64_2_148513 | 1 | 1 |  |
| 6 | c41197.graph_c0 | protein of unknown function DUF4408 | fSAESEQPSNSIGEPSR | FSAESEQPSNSIGEPSR_-64_2_155440 | 1 | 1 |  |
| 7 | c40698.graph_c0 | desiccation protectant protein lea14 homolog | gEEDGVYTYLVk | GEEDGVYTYLVK_-64_2_148549 | 1 | 1 |  |
| 8 | c39005.graph_c0 | ribulose bisphosphate carboxylase oxygenase chloroplastic | sYNLDNNMDGFYIAPAFMDk | SYNLDNNMDGFYIAPAFMDK_-64_2_172462 | 1 | 1 |  |
| 9 | c39072.graph_c0 | 21 kda | aSDFLSGLQk | ASDFLSGLQK_-64_2_291262 | 1 | 1 |  |
| 10 | c39498.graph_c0 | photosystem ii stability assembly factor chloroplastic | sISSAEDEDFNYR | SISSAEDEDFNYR_-64_2_139430 | 1 | 1 |  |
| 11 | c27213.graph_c0 | calmodulin-binding partial | dFDDVANQETEIR | DFDDVANQETEIR_-64_2_140910 | 1 | 1 |  |
| 12 | c37923.graph_c1 | trihelix transcription factor gt-2-like | gPLWEEISSAMk | GPLWEEISSAMK_-64_2_300566 | 1 | 1 |  |
| 13 | c16363.graph_c1 | Protein of unknown function DUF1191 | sLDAILQDYAYR | SLDAILQDYAYR_-64_2_132150 | 1 | 1 |  |
| 14 | c40925.graph_c0 | thylakoid lumenal protein chloroplastic | yASGTNPVTGVSTR | YASGTNPVTGVSTR_-64_2_130971 | 1 | 1 |  |
| 15 | c39244.graph_c0 | 50s ribosomal protein chloroplastic | eVQPENPNSYR | EVQPENPNSYR_-64_2_125537 | 1 | 1 |  |
| 16 | c40671.graph_c0 | proline-rich family protein | aQVSGDGQGcR | AQVSGDGQGCR_-64_2_111515 | 1 | 1 |  |
| 17 | c40052.graph_c0 | PREDICTED: uncharacterized protein LOC105160472 | nILGISSDSLLESR | NILGISSDSLLESR_-64_2_137492 | 1 | 1 |  |
| 18 | c15936.graph_c0 | probable calcium-binding protein cml36 | sIGDGQcTLEDcR | SIGDGQCTLEDCR_-64_2_138028 | 1 | 1 |  |
| 19 | c28763.graph_c1 | neurofilament heavy polypeptide isoform x2 | nIPSTSSSSR | NIPSTSSSSR_-64_2_104721 | 1 | 1 |  |
| 20 | c17618.graph_c1 | pectin methylesterase 3 | dITFQNTAGPSGNQAVALR | DITFQNTAGPSGNQAVALR_-64_2_38740 | 1 | 1 |  |
| 21 | c24853.graph_c0 | wpp domain-interacting protein 1-like | dIGkDESEPLTFDDSIR | DIGKDESEPLTFDDSIR_-64_2_45700 | 1 | 1 |  |
|  |  |  |  |  |  |  |  |
| 22 | c25447.graph_c0 | probable inactive receptor kinase at4g23740 | iLLDFIQk | ILLDFIQK_-64_2_286440 | 1 | 1 |  |
| 23 | c15385.graph_c0 | calcineurin subunit b | iELIFk | IELIFK_-64_2_262654 | 1 | 1 |  |
| 24 | c40158.graph_c0 | mannose-binding lectin | iALIGPWGGSDGENWSFk | IALIGPWGGSDGENWSFK_-64_2_167356 | 1 | 1 |  |
| 25 | c36783.graph_c1 | map3k epsilon protein kinase 1-like isoform x2 | fSDTPGDASLDDLFQPMDk | FSDTPGDASLDDLFQPMDK_-64_2_170003 | 1 | 1 |  |
| 26 | c34364.graph_c0 | grip and coiled-coil domain-containing protein 2 isoform x5 | gQLEAVNAEck | GQLEAVNAECK_-64_2_297638 | 1 | 1 |  |
| 27 | c39243.graph_c0 | 50s ribosomal protein chloroplastic | tTEEINDEIVDLk | TTEEINDEIVDLK_-64_2_155481 | 1 | 1 |  |
| 28 | c43185.graph_c0 | udp-glycosyltransferase 91a1-like | sSSDFEPEWLNLLk | SSSDFEPEWLNLLK_-64_2_160941 | 1 | 1 |  |
| 29 | c40468.graph_c0 | mitochondrial import inner membrane translocase subunit tim22-2 | gSFPEAVSSAk | GSFPEAVSSAK_-64_2_129370 | 1 | 1 |  |
| 30 | c24245.graph_c1 | type i cytoskeletal 10 | sEQPIDSNSDVTYAALR | SEQPIDSNSDVTYAALR_-64_2_36002 | 1 | 1 |  |
| 31 | c28351.graph_c0 | pyruvate decarboxylase 1 | iGGAScVQSSSVPFNSTEATLGR | IGGASCVQSSSVPFNSTEATLGR_-64_2_47129 | 1 | 1 |  |
| 32 | c44986.graph_c0 | harpin-induced family protein | tTSELDLESHNVk | TTSELDLESHNVK_-64_2_33103 | 1 | 1 |  |
| 33 | c34927.graph_c0 | isoform 1 | dSVEQEVIQGGDDR | DSVEQEVIQGGDDR_-64_2_140492 | 1 | 1 |  |
| 34 | c31639.graph_c0 | cytochrome p450 93a3-like | sVELEGELIk | SVELEGELIK_-64_2_131703 | 1 | 1 |  |
| 35 | c33588.graph_c1 | math domain-containing protein at5g43560-like | vLDGFVDADTLIIk | VLDGFVDADTLIIK_-64_2_155513 | 1 | 1. |  |
|  |  |  |  |  |  |  |  |
| 36 | c52377.graph_c0 | uncharacterized protein | iLAGLPR | ILAGLPR_-64_2_91368 | 1 | 1 |  |
| 37 | c28510.graph_c1 | cytochrome p450 cyp72a219-like | iFIMDPELIk | IFIMDPELIK_-64_2_138882 | 1 | 1 |  |
| 38 | c16195.graph_c0 | stress response protein nst1-like | qSSIWIDEVELEk | QSSIWIDEVELEK_-64_2_157965 | 1 | 1 |  |
| 39 | c42230.graph_c0 | 39s ribosomal protein mitochondrial | iVASVLFER | IVASVLFER_-64_2_104619 | 1 | 1 |  |
| 40 | c22849.graph_c0 | gpi ethanolamine phosphate transferase 1 | lVLLVADGLR | LVLLVADGLR_-64_2_106628 | 1 | 1 |  |
| 41 | c26249.graph_c0 | k+-h+ exchange-like protein | lLVFPIk | LLVFPIK_-64_2_111513 | 1 | 1 |  |
| 42 | c26884.graph_c1 | zinc finger ccch domain-containing protein 34-like | aSGGEYPER | ASGGEYPER_-64_2_100791 | 1 | 1 |  |
| 43 | c41927.graph_c0 | uncharacterized gtp-binding protein at5g64813-like | vLVVGDSGVGk | VLVVGDSGVGK_-64_2_125725 | 1 | 1 |  |
| 44 | c24245.graph_c1 | type i cytoskeletal 10 | sYEPVFAVIQIGSHQFk | SYEPVFAVIQIGSHQFK_-64_2_45920 | 1 | 1 |  |
| 45 | c32207.graph_c0 | homeobox-leucine zipper protein glabra 2 | dATNYTDEQQLR | DATNYTDEQQLR_-64_2_134052 | 1 | 1 |  |
| 46 | c29773.graph_c0 | autophagy-related protein 101 | lVLDPGESASEER | LVLDPGESASEER_-64_2_130390 | 1 | 1 |  |
| 47 | c44005.graph_c0 | fact complex subunit ssrp1 | dGVAAVLQDEEDDNVDPHLER | DGVAAVLQDEEDDNVDPHLER_-64_2_304624 | 1 | 1 |  |
| 48 | c42683.graph_c0 | rrna-processing protein efg1 | rPVESGTGNSGSNLSSNSDAR | RPVESGTGNSGSNLSSNSDAR_-64_2_42469 | 1 | 1 |  |
| 49 | c32457.graph_c0 | biogenesis of lysosome-related organelles complex 1 subunit 2 | vAEEYEGFGDVASGLR | VAEEYEGFGDVASGLR_-64_2_149809 | 1 | 1 |  |
| 50 | c49262.graph_c0 | acyl-protein thioesterase 2-like | aSSLPILLcHGk | ASSLPILLCHGK_-64_2_27073 | 1 | 1 |  |
| 51 | c41831.graph_c0 | pyruvate kinase isozyme chloroplastic-like isoform x3 | eENQQPSFPQR | EENQQPSFPQR_-64_2_127600 | 1 | 1 |  |
| 52 | c38520.graph_c0 | cyclopropane-fatty-acyl-phospholipid synthase | lTSAMAASSR | LTSAMAASSR_-64_2_102178 | 1 | 1 |  |
| 53 | c35082.graph_c0 | mediator of rna polymerase ii transcription subunit 15 isoform x2 | sDNLEENEIR | SDNLEENEIR_-64_2_117123 | 1 | 1 |  |
| 54 | c31549.graph_c0 | global transcription factor group isoform 1 | lVSELELVk | LVSELELVK_-64_2_125743 | 1 | 1 |  |
| 55 | c32250.graph_c1 | gdsl-motif lipase hydrolase family protein | vLQLQAk | VLQLQAK_-64_2_266704 | 1 | 1 |  |
| 56 | c42466.graph_c0 | rrna methyltransferase mitochondrial | nFSTETAESVTEER | NFSTETAESVTEER_-64_2_143808 | 1 | 1 |  |
| 57 | c41744.graph_c0 | 50s ribosomal protein l19- chloroplastic-like | sDPQDEAATATEVAPR | SDPQDEAATATEVAPR_-64_2_29104 | 1 | 1 |  |
| 58 | c34641.graph_c0 | peroxidase 55-like | vQQTFVTIPGTLR | VQQTFVTIPGTLR_-64_2_22288 | 1 | 1 |  |
| 59 | c42609.graph_c0 | dctp pyrophosphatase 1-like | lADIcGIDLGHAALNk | LADICGIDLGHAALNK_-64_2_39535 | 1 | 1 |  |
| 60 | c57075.graph_c0 | uncharacterized protein | lPTLLVHLR | LPTLLVHLR_-64_2_9137 | 1 | 1 |  |
| 61 | c35983.graph_c0 | transcription factor bhlh130-like | eLGESDQISSTENQQNDER | ELGESDQISSTENQQNDER_-64_2_166131 | 1 | 1 |  |
| 62 | c23226.graph_c2 | ribulose bisphosphate carboxylase oxygenase chloroplastic-like | vQLAETYLDSAALGDANR | VQLAETYLDSAALGDANR_-64_2_37172 | 1 | 1 |  |
| 63 | c40554.graph_c0 | wd repeat-containing protein lwd1 | lAIASLLEQYPNR | LAIASLLEQYPNR_-64_2_136410 | 1 | 1 |  |
| 64 | c36105.graph_c0 | had-like domain-containing protein | sDQTQQESQNEITR | SDQTQQESQNEITR_-64_2_147817 | 1 | 1 |  |
| 65 | c40106.graph_c0 | unknown | lSEETTLGSPTIEESEk | LSEETTLGSPTIEESEK_-64_2_165609 | 1 | 1 |  |
| 66 | c36087.graph_c0 | 3 -n-debenzoyl-2 -deoxytaxol n-benzoyltransferase | ayAAADHQNADPFDVITAALSTALVk | AYAAADHQNADPFDVITAALSTALVK_-64_2_174796 | 1 | 1 |  |
