# Supplementary material for: Quantitative Proteomics Analysis of Lettuce (Lactuca sativa L.) Reveals Molecular Basis-Associated Auxin and Photosynthesis with Bolting Induced by High Temperature
Source: Int J Mol Sci. 2018 Sep 28;19(10):2967. doi: 10.3390/ijms19102967 (PMC6213495; doi:10.3390/ijms19102967)
Supplement: Supplementary file 1 [file ijms-19-02967-s001.zip › ijms-336629 Supplementary/supplemental table S5.docx]

Supplemental Table S5. List of primers used in qRT-PCR

| Primer No. | Primer sequence（5-3） | Primer length |
| --- | --- | --- |
| EIX1-F | CGTAGTCGCCGTTCTCCTAT | 20 |
| EIX1-R | CCTTGCTCTCCTTCCTCAGT | 20 |
| AFP-F | AGAAGCCACCATTCCAGAGA | 20 |
| AFP-R | GGGTTCACTTCGATGACTGC | 20 |
| ADF2MC4-F | TTGGTCGTTTGGTAATGCAA | 20 |
| ADF2MC4-R | AACTCTGCGGCTTTATCGAG | 20 |
| ADF2MM -F | CCCTGTTCAATCCATCCTGA | 20 |
| ADF2MM -R | CTCAATGCCCGAGTCAATCT | 20 |
| AACT-F | TGTCAGCACCACAGTCAACA | 20 |
| AACT-R | CCAGCCACAACAACATCATT | 20 |
| CYP 71A22L-F | ATCTCACCTTTGCCACCAGA | 20 |
| CYP 71A22L-R | TTTCCACCTTGCTCTTGCTC | 20 |
| GSTL3L-F | TCAGTTTGGTGGACTTTGCTT | 21 |
| GSTL3L-R | TCTTGTTCAGTTCCGCTATCC | 21 |
| GSTL3-F | ATCCGTGACCACCTCTTTCA | 20 |
| GSTL3-R | ATCCACCAGGCTCAACTGAC | 20 |
| PDG-F | ATGGGAATCGTCGGAAATCT | 20 |
| PDG-R | CGGAGGAAGAGCAATCAAAG | 20 |
| ACO1-F | ATGGCGAATCCGTAATTTCA | 20 |
| ACO1-R | GATTCAAATCTCGGCCCTTT | 20 |
| STPK -F | CAAAGCAGGGAGTTCGTGA | 19 |
| STPK -R | CGCAGAGTCAAGGCTGTTATT | 21 |
| PSTPK-F | GAAGCCTCAAGAAGCATTGG | 20 |
| PSTPK-R | CATGGGCTTCAGTTTCCATT | 20 |
| NPR1-F | TCTTGCTCTTGATACTCTAT | 20 |
| NPR1-R | ATTGCTAATTGCTTCTGG | 18 |
| PLRLSTPKRIX1-F | GCACACGAGTAGCTGGAACA | 20 |
| PLRLSTPKRIX1-R | TCACAATTTCCAAAGCCACA | 20 |
| actin-F | GTGAGTGAAGAAGGGCAATG | 21 |
| actin-R | CACTTTCAACCCGATTCACC | 20 |
