# Supplementary figures and images for: Quantitative Proteomics Analysis of Lettuce (Lactuca sativa L.) Reveals Molecular Basis-Associated Auxin and Photosynthesis with Bolting Induced by High Temperature
Source: Int J Mol Sci. 2018 Sep 28;19(10):2967. doi: 10.3390/ijms19102967 (PMC6213495; doi:10.3390/ijms19102967)

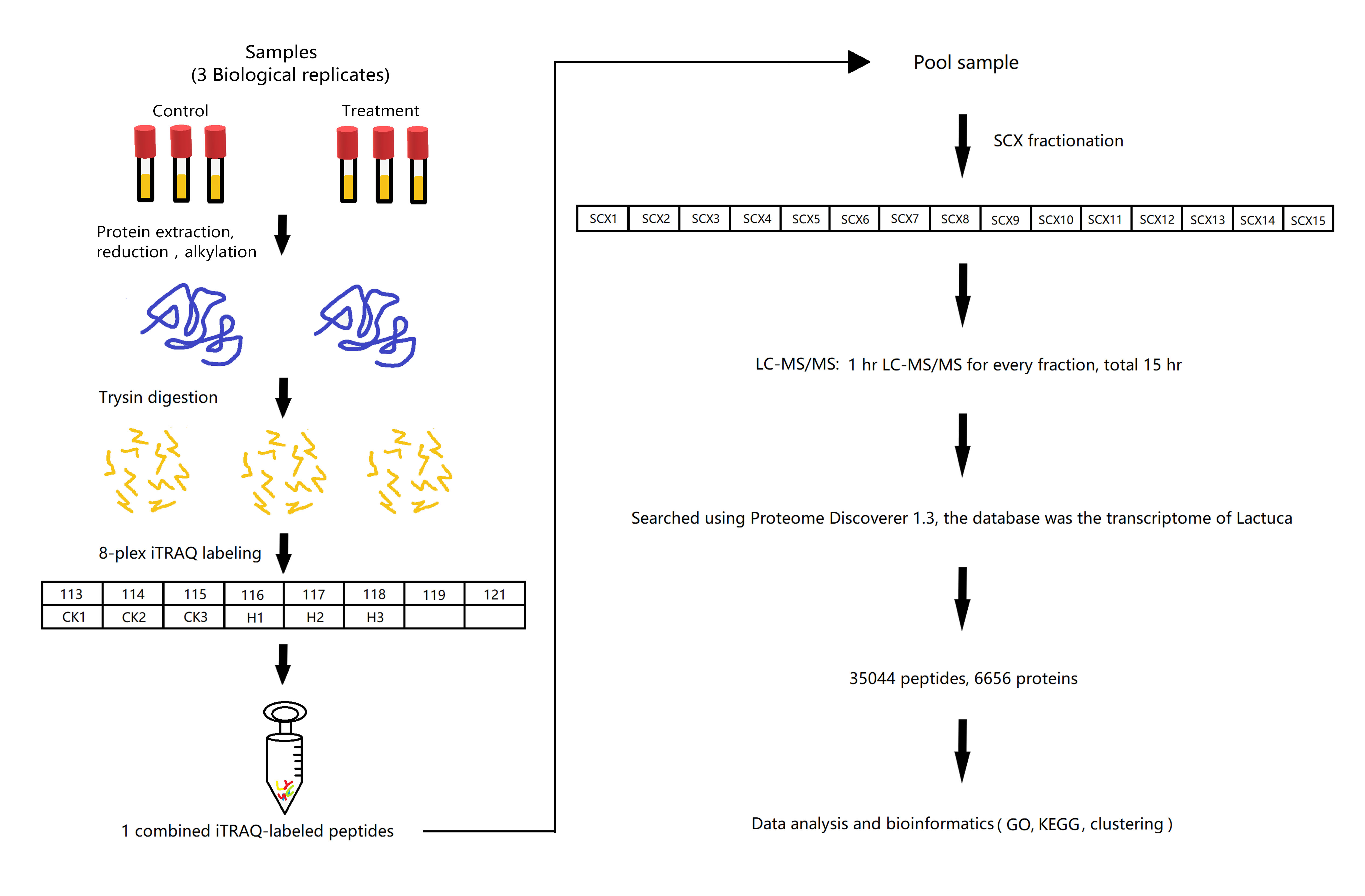

Supplement: Supplementary file 1 [file ijms-19-02967-s001.zip › ijms-336629 Supplementary/Supplemental Figure 1.jpg]
